# TOR3A represses type I interferon production and limits viral clearance during respiratory syncytial virus infection

**DOI:** 10.1080/22221751.2026.2637961

**Published:** 2026-02-25

**Authors:** Xiaoping Li, Zhengrong Chen, Mengyun Wu, Peijie Zhu, Guodong Qiao, Jiaoyang Li, Yunfei Ye, Jiamin Cai, Ying Zhou, Xiaoqiu Dai, Yufeng Wang, Cancheng Li, Jiaqi Huang, Ji Zhou, Fei Xu, Chensheng Tan, Yu Shao, Xiu Gao, Jingjing Hu, Xuena Xu, Chunsheng Dong, Chuangli Hao, Yi Yang, Jinping Zhang

**Affiliations:** a The First Affiliated Hospital, Institutes of Biology and Medical Sciences, Suzhou Medical College, Soochow University, Suzhou, People’s Republic of China; b Department of Clinical Laboratory, The First Affiliated Hospital of Soochow University, Suzhou, People’s Republic of China; c Institutes of Biology and Medical Sciences, Suzhou University, Suzhou, People’s Republic of China; d Department of Respiratory Medicine, Children's Hospital of Soochow University, Suzhou, People’s Republic of China; e Department of Respiratory Medicine, Second Affiliated Hospital of Zhejiang University School of Medicine, Hangzhou, People’s Republic of China

**Keywords:** RSV, macrophages, TOR3A, RIG-I, ubiquitination

## Abstract

Type I interferons (IFN-I) are essential for antiviral immunity, and precise regulation of IFN-I production is crucial to balance viral clearance and immunopathology. Here, we demonstrate that the interferon-stimulated gene *TOR3A* negatively regulates type I IFN signalling during respiratory syncytial virus (RSV) infection. *TOR3A* expression was upregulated in macrophages and RSV-infected patients, and its deficiency enhanced antiviral responses, leading to reduced viral load. Mechanistically, RSV infection induced *TOR3A* expression through the IFN-STAT1 pathway, which in turn suppressed IFN-I production. Furthermore, TOR3A recruited the E3 ubiquitin ligase STUB1 to mediate K48-linked ubiquitination and proteasomal degradation of RIG-I at lysine 146, thereby promoting RSV immune evasion. Our study identifies TOR3A as a novel suppressor of antiviral immunity and uncovers a mechanism by which RSV exploits host ISGs to dampen IFN-I responses, providing new insights into viral pathogenesis and potential therapeutic strategies.

## Introduction

In 1956, Morris and associates first isolated human respiratory syncytial virus (RSV) from chimpanzees with upper respiratory tract infections. They identified the virus as the primary cause of most epidemic bronchiolitis in infants and young children [1]. RSV is also a major cause of severe acute lower respiratory tract infections, impacting infants, young children, the elderly, and immunocompromised individuals worldwide [2]. RSV infection is prevalent globally, infecting approximately 70% of infants by their first year and nearly all infants under two [3,4]. In 2019, about 33 million infants under five globally had RSV-related acute lower respiratory tract infections, with 3.6 million hospitalized and 100,000 fatalities [5]. Vaccines developed by GSK, Pfizer, and Moderna, approved by the Food and Drug Administration (FDA) in 2023–2024, mainly target the elderly and pregnant women [6]. At present, no vaccines are available for infants and young children. Additionally, the aforementioned vaccines may pose safety risks [7]. Symptomatic supportive treatment is currently the main strategy in clinical practice [8]. Therefore, exploring the molecular pathogenesis of RSV infection is crucial for identifying new intervention targets [9].

RSV pathogenesis involves a complex interplay between the virus and host cells, viral replication, and host immune response [3,8,10]. IFN-I is a key innate immune barrier to viral infections [11,12]. In RSV, IFN-I accelerate virus clearance, boost protective immune response, and reduce disease severity [13]. Various types of cells, including dendritic cells, epithelial cells, and alveolar macrophages (AMs), can produce IFN-I during RSV infection. Goritzka et al. found that AMs are the main source of IFN-I in the lungs of RSV-infected mice [14]. The production of IFN-I by AMs during RSV infection mainly depends on the retinoic acid-inducible gene I (RIG-I)-like receptor (RLR) signalling pathway. AMs recognize the viral nucleic acid of RSV through RIG-I, thereby binding to and promoting the downstream protein mitochondrial antiviral signalling (MAVS) to form prion protein-like aggregation on mitochondria [15]. MAVS induces the phosphorylation of downstream TANK-binding kinase 1 (TBK1) and interferon regulatory factor 3 (IRF3) [16,17]. Phosphorylated IRF3 dimerizes and translocates to the nucleus to initiate transcription of interferon genes, producing antiviral effects [18,19]. The production of IFN-I is regulated by RSV and host factors; We have conducted some interesting research in this area [9,20], however, the specific mechanism still needs to be understood.

The human genome encodes four types of Torsin, including TorsinA, TorsinB, Torsin2A, and Torsin3A [21]. This family is part of the AAA + superfamily of proteins associated with various cellular activities [21]. Torsin is the only AAA + protein present in the nuclear envelope (NE) and endoplasmic reticulum (ER) [22]. Torsins may act as molecular chaperones by affecting the conformation of target proteins [23]. Torsins have also been implicated in other cell biological processes, including lipid metabolism [24], endoplasmic reticulum oxidation reduction monitoring [25], formation of nuclear pore complexes [26]. Torsin family 3 member A (*TOR3A*), also known as ATP-dependent interferon-responsive protein (ADIR) [27], is a member of the Torsin ATPase family. A previous study found that the expression of TOR3A increased 5–15-fold in the oral cavity, spleen, and liver tissues of mice orally or intraperitoneally injected with IFN-α [27]. This suggests that *TOR3A* may belong to interferon-stimulated genes (ISGs). However, the role of TOR3A in innate immunity against RSV is yet to be deciphered.

Herein, we performed a mass spectrometry analysis, revealing a significant increase in TOR3A in RSV-infected RAW264.7 cells compared with control cells. Our research also demonstrated that the signal transducer and activator of transcription 1 (STAT1), downstream of the IFN-I signalling pathway, bound to the TOR3A promoter after RSV infection, thereby increasing *TOR3A* expression. Clinical studies show that *TOR3A* mRNA expression in peripheral blood mononuclear cells (PBMCs) of RSV-infected patients is significantly higher than the control group, and positively correlated with infection severity and viral copy number. ROC analysis suggests TOR3A may predict RSV infection and related bronchiolitis. Further analyses revealed that TOR3A overexpression inhibited IFN-I production and promoted RSV virus replication; TOR3A knockout significantly reduced lung inflammation in RSV-infected mice. Moreover, TOR3A facilitated the interaction between RIG-I and the E3 ubiquitin ligase STUB1, promoting K48-linked polyubiquitination of RIG-I by STUB1 at the K146 site.

## Results

### STAT1 regulates the elevation of TOR3A expression during RSV infection

To study RSV-host interactions, RAW264.7 cells were infected with mCherry-labeled RSV L19 (RSV-mCherry). Next, mass spectrometry was performed to profile global protein abundance changes after RSV infection and to identify pathways and molecules enriched among differentially abundant proteins in RSV-infected RAW264.7 cells (Figure 1A). Cluster analysis of the proteomics data showed that multiple ISGs displayed increased protein abundance after RSV infection (Figure 1B). Previous studies have shown that ISGs such as *ISG15* [28] and *DDX54* [29] are implicated in the regulation of viral replication. During our analysis of the RSV infection dataset, we noticed a marked upregulation of TOR3A protein level (Figure 1B). Despite its known induction by IFN-I [27], the regulation of TOR3A during viral infection remains unclear. KEGG pathway analysis indicated that proteins associated with multiple infection-related signalling pathways were significantly enriched among the differentially abundant proteins after RSV infection (Figure 1C). GO enrichment analysis revealed significant enrichment of terms related to ubiquitin ligase activity and chaperone binding after RSV infection (Figure 1D). RT-qPCR and Western blot were performed to verify the mass spectrometry results. Results showed that *Ifnb mRNA* expression was significantly increased in RSV-infected RAW264.7 cells, indicating induction of the IFN-I signalling (Figure 1E). Further analysis showed that *Tor3a expression* was higher in RSV-infected RAW264.7 cells compared with the control group, both at the RNA level (Figure 1F) and the protein level (Figure 1G). RAW264.7 cells were then stimulated with the RNA virus mimic Poly(I:C), and the results were consistent with RSV infection (Fig. S1A-C). Subsequently, mouse primary PMs were infected with RSV, and results showed that RSV upregulated *Ifnb* and *Tor3a* expression in PMs (Fig. S1D-F). Similar results were observed in bone-marrow-derived macrophages (BMDMs) (Fig. S1G-I). Next, we infected the human monocyte cell line THP-1 with RSV and the result showed that TOR3A protein expression was upregulated (Fig. S1J), indicating that RSV elevates TOR3A levels in both humans and mice. Next, we explored whether TOR3A upregulation in RSV-infected macrophages was specifically induced by RSV. RAW264.7 cells were infected with RNA virus vesicular stomatitis virus (VSV), Sendai virus (SeV) and DNA virus herpes simplex virus type 1 (HSV-1). RT-qPCR results showed that these viruses upregulated *Ifnb and Tor3a* mRNA expression levels (Fig. S2A-F). Both DNA viruses and RNA viruses can induce *Ifnb* and *Tor3a* expression, further supporting that *Tor3a* is an interferon-stimulated gene.

**← Figure 1.**
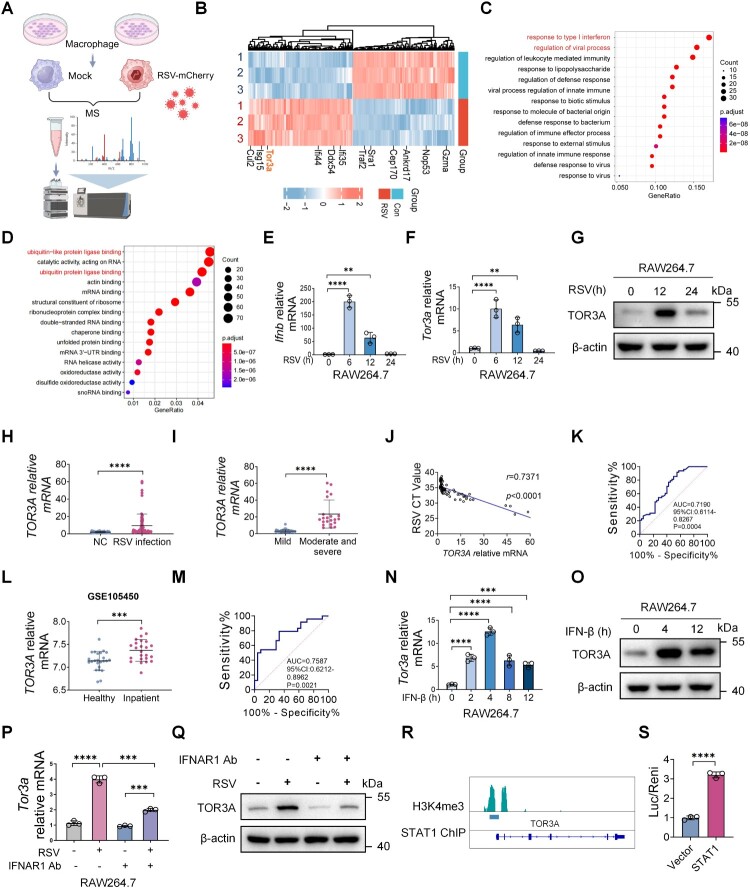
The upregulation of TOR3A during RSV infection is facilitated by STAT1. (**A**) Flowchart overview of the infection of RAW264.7 cells with RSV followed by mass spectrometry analysis. RAW264.7 cells were infected with RSV (MOI = 1) for 12 h, with 2% DMEM (RSV solvent) as control. (**B**) Whole protein profiles of RAW264.7 cells after 12-hour RSV infection were analyzed. Cluster analysis identified differentially expressed interferon-stimulated genes (ISGs) proteins between RSV-infected and control groups. (**C**) KEGG analysis demonstrated activation of multiple infection-related signalling pathways following RSV infection. (**D**) GO enrichment analysis showing significant activation of cellular functions in response to RSV infection. (**E**-**F**) RT-qPCR analysis of *Ifnb* and *Tor3a* mRNA levels in RAW264.7 cells at 0, 6, 12, and 24 h post-RSV infection. (**G**) Western blot analysis of TOR3A protein expression in RAW264.7 cells at indicated time points (0, 12, 24 h) post-RSV infection. (**H**) RT-qPCR analysis of *TOR3A* mRNA levels in PBMCs from hospitalized RSV-bronchiolitis patients (n = 70) and controls (n = 40). (**I**) RT-qPCR analysis of *TOR3A* expression in PBMCs from RSV-infected children with mild (n = 48) or moderate to severe (n = 22) disease. (**J**) Analysis of the correlation between *TOR3A* mRNA levels and RSV viral copy number. (**K**) ROC analysis was performed to evaluate the predictive value of *TOR3A* expression for bronchiolitis in RSV-infected children. (**L**) Analysis of *TOR3A* mRNA levels from peripheral blood transcriptomic data (GSE188424) in hospitalized RSV-infected children (n = 24) versus age-matched healthy controls (n = 24). (**M**) ROC curve analysis of the predictive value of TOR3A for RSV infection. (**N-O**) RAW264.7 cells were stimulated with IFN-β at a working concentration of 1000 U/ml. The mRNA and protein levels of TOR3A were measured by RT-qPCR and Western blot, respectively. (**P-Q**) RAW264.7 cells were pre-incubated with an IFNAR1-specific blocking antibody (100 ng/mL) for 2 h prior to RSV infection (12 h). The mRNA and protein levels of TOR3A were then measured by RT-qPCR and Western blot, respectively. (**R**) ChIP analysis revealed that STAT1 binds to the *Tor3a* promoter. (**S**) HEK293 T cells were co-transfected for 24 h with a luciferase reporter plasmid driven by the TOR3A promoter, a STAT1 expression plasmid, and empty vector, followed by a dual-luciferase reporter assay. Data are representative of three independent experiments and presented as mean ± SD. Statistical significance was determined by Student's t-test (H, I, L, and S), one-way ANOVA followed by Dunett's multiple comparisons test (E, F, N, and P). ns: no significance, *P *<* 0.05, **P *<* 0.01, ***P *<* 0.001, ****P *<* *0.0001.*

To determine if RSV induces *Tor3a* expression in vivo, C57BL/6J mice were intranasally infected with RSV. RSV-F mRNA in lung tissue was significantly increased at day 3 post-infection (Fig. S2G), and Tor3a mRNA was also increased (Fig. S2H), indicating that RSV infection is associated with elevated Tor3a expression in *vivo*. AMs are the main source of IFN-I in the lungs of RSV-infected mice [14], and the increase in *Tor3a* is strongly correlated with the increase in IFN-I. Hence, we speculate that AMs are likely to play an important role in this process. Next, single-cell suspensions from lung tissue after RSV infection of C57BL/6J mice were stained to determine whether the increase in *Tor3a* after RSV infection is cell-specific. The results showed that RSV infection significantly upregulated the expression of TOR3A protein in AMs, while no significant difference in TOR3A expression was found in other cell populations (Fig. S2I). In addition, peripheral blood samples from pediatric patients with RSV infection (Table S1) were analyzed to ascertain the role of TOR3A in the host’s defense against RSV infection. We found that *TOR3A* mRNA expression in PBMCs was elevated in patients compared to the control group (Figure 1H). Among these infected patients, *TOR3A* mRNA was significantly increased in PBMCs from 22 cases of moderate and severe infection in comparison with the 48 cases of mild infection (Figure 1I). Furthermore, *TOR3A* expression was positively correlated with the viral copy number of RSV (Figure 1J), suggesting an association with disease burden in this cohort. ROC analysis showed that TOR3A expression exhibited moderate discriminative performance between groups in this cohort (AUC = 0.719; Figure 1 K). Consistently, analysis of the public dataset GSE164015 indicated higher *TOR3A* expression in hospitalized RSV patients than in healthy controls, with similar discriminative performance (AUC = 0.7587; Figure 1L-M). Taken together, these data suggest that TOR3A expression in PBMCs is associated with RSV disease severity in this cohort; however, this association may be influenced by potential confounders such as changes in leukocyte composition and variability in sampling time relative to symptom onset and treatment.

Previous studies [27] have shown that IFN-I can induce the expression of *Tor3a* (ADIR); however, whether RSV infection similarly upregulates *Tor3a* remains to be determined. Therefore, we wanted to investigate whether the elevation of TOR3A after RSV infection is regulated downstream by IFN-I. Consistent with the reported data, *Tor3a* mRNA and TOR3A protein levels increased when RAW264.7 cells were stimulated with IFN-β (Figure 1N-O). To explore whether the increase in *Tor3a*. expression during RSV infection is also dependent on the IFN-I downstream pathway, IFNAR1-specific blocking antibodies were used to block the IFN-I pathway in RAW264.7 cells, followed by RSV infection. Results showed that *Tor3a* induction by RSV infection was significantly inhibited in the antibody-blocked group (Figure 1P-Q). These results indicate that the increase in *Tor3a* is mediated by the IFN-I pathway. It is known that phosphorylated STAT1 and STAT2, together with IRF9, form the ISGF3 complex downstream of IFN-I signalling [30], which translocates into the nucleus and induces the transcription of ISGs such as *Ifit1* and *Rsad2* (viperin), ultimately exerting a series of biological functions. Therefore, is *Tor3a* transcription regulated by ISGF3? According to the ChIP results [31] from the Cistrome Data Browser and the transcription factor binding site information from ENCODE3, STAT1 was shown to bind to the promoter of *TOR3A* (human) and *Tor3a* (mouse) in macrophages (Figure 1R). Therefore, a dual-luciferase reporter gene assay was employed to test whether STAT1 exerts a regulatory effect on *Tor3a*. The findings demonstrated that STAT1 can directly stimulate *Tor3a* promoter activity, leading to enhanced *Tor3a* expression (Figure 1S). Given that Tor3a is an interferon-stimulated gene and our data support an IFN-I-STAT1 axis driving Tor3a induction during RSV infection, we next examined whether IFN-III interferon (IFN-λ) can also regulate TOR3A expression in macrophages, with IFN-β included as a positive control. Notably, IFN-λ responsiveness differed between macrophage models. In RAW264.7 cells, IFN-λ failed to induce TOR3A and elicited minimal changes in IFNLR1/IL10RB expression (Fig. S2J–M). In contrast, in primary BMDMs, IFN-λ significantly increased TOR3A expression together with induction of IFNLR1/IL10RB (Fig. S2N–Q). This cell-type–dependent effect is consistent with prior evidence [32] that IFN-λ responsiveness is shaped by the availability of IFNLR1 and can be acquired during monocyte-to-macrophage differentiation [33]. Indeed, M-CSF-driven macrophage differentiation has been reported to markedly upregulate IFNLR1, whereas IL10RB remains largely unchanged, thereby conferring IFN-λ responsiveness. Mechanistically, IFN-λ signals through the IFNLR1/IL10RB receptor complex to activate JAK–STAT and ISGF3 (STAT1/STAT2/IRF9) [34,35], leading to ISG induction; accordingly, BMDMs unlike RAW264.7 cells display robust TOR3A upregulation upon IFN-λ stimulation.

### TOR3A suppresses IFN-I production and promotes RSV replication *in vitro*

To investigate TOR3A's role in antiviral responses, RAW264.7 cells were stably transduced with TOR3A-targeting or control shRNA lentivirus. TOR3A knockdown was confirmed at both mRNA and protein levels (Figure 2A-B). TOR3A-downregulated RAW264.7 cells showed decreased RSV-F mRNA expression (Figure 2C) and mCherry-positive cells (Figure 2D) following RSV-mCherry infection. IFN-I is one of the main innate immune barriers to viral infection. To assess whether TOR3A affects RSV infection via IFN-I regulation, we measured *Ifnb* and *Isg15* expression and found it significantly increased in shTOR3A cells compared to shNC controls (Figure 2E-F). To further establish a causal role for TOR3A in restraining IFN-I responses during RSV infection, we performed a rescue experiment and found that re-expression of TOR3A (Figure 2G) in TOR3A-knockdown cells significantly reduced *Ifnb* and *Isg15* expression compared with empty-vector controls (Figure 2H-I). Consistent with this, IFN-I production–defective Vero cells were infected with RSV or VSV, and TOR3A overexpression did not enhance viral replication (Fig. S3A-B), suggesting that the antiviral effect of TOR3A is largely dependent on its modulation of the IFN-I response.

**← Figure 2.**
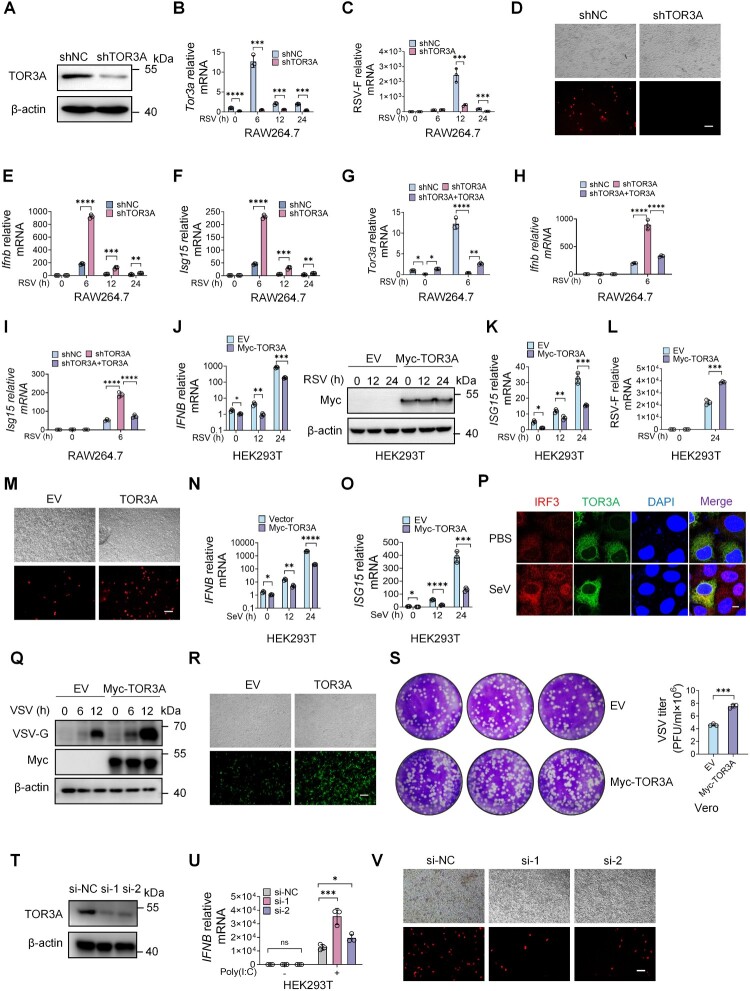
TOR3A inhibits IFN-I production and facilitates RSV replication *in vitro*. (**A**) TOR3A expression was detected by Western blot in RAW264.7 cells stably transfected with shTOR3A. (**B-C**) RT-qPCR analysis of *Tor3a* and RSV-F mRNA levels in control and shTOR3A cells at 0, 6, 12, and 24 h post-RSV infection. (**D**) After 24 h of RSV-mCherry infection, RSV replication was visualized by fluorescence microscopy in shTOR3A and control cells (scale bar, 100 μm; top: bright field image; bottom: fluorescence image). (**E-F**) RT-qPCR analysis of *Ifnb* and *Isg15* mRNA levels in control and shTOR3A cells at 0, 6, 12, and 24 h post-RSV infection. (**G-I**) RAW264.7 cells with TOR3A knockdown (shTOR3A) were transfected with an empty vector (EV) or a TOR3A expression plasmid (shTOR3A + TOR3A), followed by RSV infection for 6 h. *Tor3a*, *Ifnb*, and *Isg15* mRNA levels were measured by RT-qPCR. (**J-K**) RT-qPCR analysis of *IFNB* and *ISG15* mRNA levels in HEK293 T cells at 12 and 24 h post-RSV infection, following 24 h of transfection with Myc-TOR3A. (**L**) RT-qPCR analysis of RSV-F mRNA levels in HEK293 T cells at 24 h post-RSV infection, following 24 h of transfection with Myc-TOR3A. (**M**) Fluorescence microscopy analysis of RSV infection in HEK293 T cells at 24 h post-infection, following 24 h of transfection with Myc-TOR3A. Scale bar, 100 μm. (**N-O**) RT-qPCR analysis of *IFNB* and *ISG15* mRNA levels in HEK293 T cells at 12 and 24 h post-SeV infection, following transfection with Myc-TOR3A for 24 h. (**P**) After overexpressing Myc-TOR3A for 24 h in HeLa cells, the cells were infected with SeV for 12 h and stained with IRF3 (red) and Myc (green) antibodies (scale bar, 10 μm). (**Q)** Western blot analysis of VSV-G protein expression at 6 and 12 h post-VSV-GFP infection in HEK293 T cells transfected with Myc-TOR3A for 24 h (**R**) After transfecting HEK293 T cells with Myc-TOR3A for 24 h, the cells were infected with VSV-GFP for 12 h, and the replication of VSV-GFP was visualized by fluorescence microscopy (scale bar, 100 μm). (**S**) After HEK293 T cells were transfected with Myc-TOR3A for 24 h, they were infected with VSV for 24 h. The virus-containing supernatant was then collected for plaque assay, and the results were subjected to statistical analysis. (**T**) TOR3A knockdown efficiency was confirmed by Western blot in HEK293 T cells transfected with si-NC or siTOR3A (si-1, si-2) for 48 h. (**U**) RT-qPCR analysis of *IFNB* mRNA levels in HEK293 T cells after 12 h of Poly(I:C) (1 μg/mL) transfection, following 36 h of TOR3A siRNA transfection. (**V**) Fluorescence microscopy of RSV replication in HEK293 T cells at 24 h post-infection, following 36 h of transfection with TOR3A siRNA. Scale bar, 100 μm. Data are representative of three independent experiments and presented as mean ± SD. Statistical significance was determined by Student's t-test (B, C, E, F, J, K, L, N, O and S), two-way ANOVA followed by Sidak's multiple comparisons test (G, H, I and U). ns: no significance, *P *<* 0.05, **P *<* 0.01, ***P *<* 0.001, ****P *<* *0.0001.*

To further confirm TOR3A’s role, we overexpressed it in HEK293 T cells and found that *IFNB* and *ISG15* mRNA levels were significantly reduced following L19 RSV infection (Figure 2J-K). Additionally, RSV-F mRNA levels and the fluorescence of m-cherry were higher in TOR3A-overexpressing cells than in control cells (Figure 2L-M). These data further demonstrate that TOR3A can suppress IFN-I production and promote RSV replication *in vitro*. Next, we replaced RSV with virus mimics or other commonly used model virus strains SeV and VSV to explore whether TOR3A exerts its function in a pathogen-specific manner. RT-qPCR results showed that TOR3A overexpression, Poly (I: C) transfection (Fig. S3C-D), or SeV infection (Figure 2N-O) significantly decreased mRNA levels of *IFNB* and *ISG15* in HEK293 T cells. Furthermore, we explored whether TOR3A inhibits the production of IFN-I by affecting the nuclear import of IRF3. Confocal results showed that TOR3A inhibited the nuclear import of transcription factor IRF3 in the IFN-I pathway (Figure 2P, S3E). HEK293 T cells were transfected with TOR3A, followed by VSV-green fluorescent protein (GFP) infection to determine whether the replication of other RNA model viruses such as VSV is also regulated by TOR3A. Western blot, fluorescence microscope, and virus plaque assays were used to detect the viral load and titre of VSV. The results showed that the viral load and plaques were significantly higher in the overexpression group than in the control group (Figure 2Q-S). Next, TOR3A was knocked down in HEK293 T, and VSV-glycoprotein (VSV-G) was detected by Western blot after VSV-GFP infection. The results showed that VSV-G decreased significantly in the TOR3A knockdown group (Fig. S3F). Virus plaque experiments also showed the VSV titre decreased significantly after TOR3A knockdown (Fig. S3G). Further Western blot results demonstrated that siRNA effectively knocked down TOR3A in HEK293 T cells (Figure 2 T). HEK293 T cells transfected with TOR3A siRNA and RNA virus mimic Poly(I:C) showed significantly elevated *IFNB* levels by RT-qPCR compared with siNC group (Figure 2U). Meanwhile, the fluorescence intensity of m-cherry was much lower in L19 RSV-infected cells than in control cells (Figure 2 V). Taken together, these data indicate that TOR3A knockdown effectively inhibits the replication of RSV. Moreover, TOR3A functions in a non-pathogen-specific manner, suggesting its broad-spectrum role in innate immunity.

### TOR3A deficiency promotes IFN-I production and protects mice against viral infection *in vivo*

To further verify the role of TOR3A in innate immunity, the TOR3A KO mouse model was constructed by a CRISPR/Cas9 technique (Fig. S4A). TOR3A KO mice were viable and normal in size and showed no gross physiological or behavioural abnormalities (data not shown). Genotyping, RT-qPCR, and Western blot results revealed that the TOR3A KO mouse model was successfully established (Fig. S4B-D). Furthermore, Poly(I:C) or RSV was used to stimulate primary PMs of wild-type (WT) and TOR3A KO mice to confirm the above *in vitro* data. RT-qPCR results showed that the level of *Ifnb* in PMs was significantly higher in KO mice than in the WT group (Fig. S4E-F). The expression of the RSV-F protein was significantly reduced in the KO group (Fig. S4G), suggesting that TOR3A KO enhances the innate antiviral response of macrophages against RSV. To determine whether TOR3A regulation of the IFN-I pathway is specific to the RLR pathway, RIG-I specific agonist 3phpRNA and cyclic GMP-AMP synthase-stimulator of interferon genes (cGAS-STING) pathway specific agonist DMXAA were used to stimulate WT and KO PMs, respectively, and *Ifnb* expression was detected. The results revealed that both agonists elevated *Ifnb* expression in TOR3A KO compared with WT PMs (Fig. S4H-I). TOR3A may regulate both the RLR and cGAS-STING pathways. RSV belongs to RNA viruses that primarily activate the RLR pathway. Therefore, this study focuses on the regulation of the RLR pathway by TOR3A.

**Figure 3.**
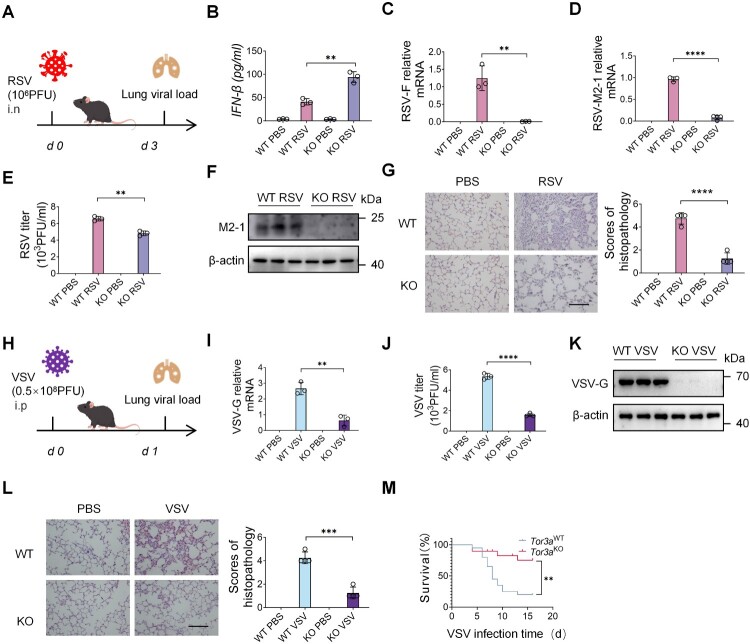
TOR3A deficiency augments IFN-I production and restrains virus replication *in vivo*. (**A**) Establishment of an intranasal RSV infection model in mice. (**B**) ELISA of serum IFN-β in WT versus TOR3A KO mice post-RSV infection (1 × 10⁶ PFU, i.n.). (**C-D**) RT-qPCR analysis of RSV-F and RSV-M2-1 mRNA levels in the lungs of RSV-infected WT and TOR3A KO mice (n = 3 per group). (**E**) Plaque assay of RSV titres in lung homogenate supernatants from RSV-infected WT and TOR3A KO mice. (**F**) Western blot analysis of M2-1 protein expression in lung tissues from RSV-infected WT and TOR3A KO mice. (**G**) H&E staining and pathological scoring of lung sections from PBS- or RSV-treated WT and TOR3A KO mice. Scale bar, 100 μm. (**H**) Establishment of a VSV intraperitoneal infection model in mice. (**I**) RT-qPCR analysis of VSV load in the lungs of WT and TOR3A KO mice (n = 3 per group) after infection (4 × 10⁸ PFU). (**J**) Plaque assay of VSV titres in lung homogenate supernatants from VSV-infected WT and TOR3A KO mice. (**K**) Western blot analysis of VSV-G protein expression in lung tissues from VSV-infected WT and TOR3A KO mice. (L) H&E staining and histopathological scoring of lung sections from PBS- or VSV-treated WT and TOR3A KO mice. Scale bar, 100 μm. (M) Survival of WT and TOR3A KO mice (n = 20 per group) after intraperitoneal infection with VSV (4 × 10⁸ PFU). Data are representative of three independent experiments and presented as mean ± SD. Statistical significance was determined by Student's t-test (B, C, D, E, G, I, J, and K) or survival curve (L) followed by Log-rank (Mantel-Cox) test. ns: no significance, *P *<* 0.05, **P *<* 0.01, ***P *<* 0.001, ****P *<* *0.0001.*

To further analyze the function of TOR3A in innate immunity *in vivo*, WT and TOR3A KO mice were infected intranasally with RSV and then sacrificed after 3 days for analysis (Figure 3A). ELISA results showed that IFN-β level was significantly higher in the serum of TOR3A KO than control mice (Figure 3B). RSV-F and RSV-M2-1 mRNA levels (Figure 3C-D), RSV titres (Figure 3E), and the M2–1 protein level (Figure 3F) in the lungs of TOR3A KO mice were significantly lower than those of WT mice, suggesting that TOR3A KO inhibits RSV replication. HE staining showed that the alveolar walls of RSV-infected WT mice were thickened, and that these mice exhibited notable infiltration of inflammatory cells and inflammatory exudation in the alveoli. In contrast, the infiltration of inflammatory cells was less in the KO group after RSV infection (Figure 3G). Collectively, these data indicate that TOR3A KO reduces RSV disease severity.

To explore whether TOR3A exerts its function in a pathogen-specific manner, WT and TOR3A KO mice were intraperitoneally infected with WT VSV, and mice were sacrificed after 24 h for analysis (Figure 3H). RT-qPCR results showed that the VSV load in the lungs was significantly lower in TOR3A KO than in WT mice (Figure 3I). VSV titres (Figure 3J), and the VSV-G protein level (Figure 3 K) in the lungs of TOR3A KO mice were significantly lower than those of WT mice. HE staining showed that the inflammatory cell infiltration and immune injury in the lungs were lighter in VSV-infected TOR3A KO than in WT mice (Figure 3L). Furthermore, TOR3A KO mice showed lower mortality rates than WT mice after intranasal infection with VSV (Figure 3M). These results are consistent with those observed in RSV-infected mice, which suggests that TOR3A KO can enhance the antiviral response and protect mice against virus infection in a non-pathogen-specific manner.

### TOR3A binds to and inhibits RIG-I in the IFN-I pathway

**Figure 4.**
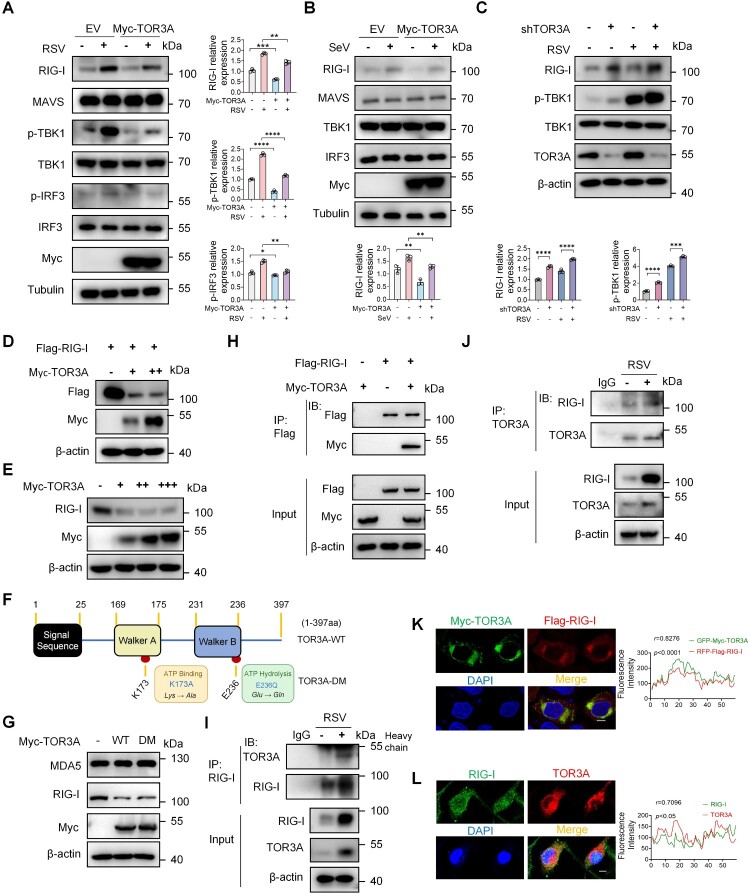
TOR3A targets RIG-I and inhibits its function in the IFN-I pathway. (**A**) Western blot analysis of IFN-I pathway proteins in HeLa cells after 12 h RSV infection, following 24 h transfection with Myc-TOR3A or empty vector (EV). (**B**) Western blot analysis of IFN-I pathway proteins in HeLa cells after 12 h SeV infection, following 24 h transfection with Myc-TOR3A or empty vector (EV). (**C**) Western blot analysis of RIG-I, p-TBK1, TBK1, and TOR3A in shTOR3A and control RAW264.7 cells at 12 h post-RSV infection. (**D**) Western blot analysis of Flag-RIG-I and gradient Myc-TOR3A expression in HEK293 T cells after 48 h of co-transfection. (**E**) Western blot analysis of RIG-I protein levels in HeLa cells transfected with increasing amounts of Myc-TOR3A for 48 h. (**F**) Schematic diagram of TOR3A domains and the ATPase double mutant TOR3A-DM (K173A/E236Q). (**G**) Western blot analysis of RIG-I and MDA5 in HeLa cells after 48 h transfection with wild-type (WT) or double mutant (DM) Myc-TOR3A. (**H**) Co-IP analysis of the interaction between Myc-TOR3A and Flag-RIG-I in HEK293 T cells after 48 h of co-transfection. (**I**) Endogenous Co-IP of TOR3A and RIG-I interaction in RAW264.7 cells after 12 h of RSV infection. (**J**) Endogenous Co-IP of TOR3A and RIG-I interaction in THP-1 cells after 12 h of RSV infection. (**K**) Co-localization of Myc-TOR3A and Flag-RIG-I in HEK293 T cells after 48 h co-transfection. Cells were immunostained for Myc (green) and Flag (red) and imaged by confocal microscopy. Co-localization was quantified by Pearson's correlation coefficient (r). Scale bar, 10 μm. (**L**) Co-localization of endogenous RIG-I (green) and TOR3A (red) in mouse primary PMs at 6 h post-SeV infection. Images were acquired by confocal microscopy, and co-localization was quantified using Pearson's correlation coefficient (r). Scale bar, 25 μm. Data are representative of three independent experiments and presented as mean ± SD. Statistical significance was determined by Student's t-test (A, B, and C). ns: no significance, *P *<* 0.05, **P *<* 0.01, ***P *<* 0.001, ****P *<* *0.0001.*

Upon sensing RSV RNA, RIG-I-like receptors (RLRs) signal via MAVS to activate the downstream kinases TBK1 and IKKϵ, which phosphorylate and activate IRF3, thereby promoting IFN-I induction [36]. To determine whether TOR3A regulates key components of the IFN-I pathway, HeLa cells were transfected with overexpressed Myc-TOR3A and empty plasmid and then infected with RSV and SeV. It was found that only the protein level of endogenous RIG-I was significantly reduced (Figure 4A-B). Furthermore, Western blot results showed that p-TBK1 and p-IRF3 levels were significantly reduced and only the protein level of RIG-I was significantly reduced after TOR3A overexpression with RSV infection (Figure 4A), suggesting that TOR3A targets and inhibits RIG-I. To further verify this result, TOR3A was knocked down in RAW264.7 cells, followed by RSV infection. Western blot results showed that RIG-I and p-TBK1 levels were significantly higher in the shTOR3A group than in the shNC group, whereas the total amount of TBK1 remained unchanged (Figure 4C). In addition, exogenous TOR3A downregulated the protein level of exogenous and endogenous RIG-I in HEK293 T and HeLa cells, respectively (Figure 4D-E). To determine whether TOR3A-mediated inhibition of RIG-I depends on its ATPase activity, we introduced motif-conserved mutations in the TOR3A ATPase domain: K173A (Walker A/P-loop; predicted ATP-binding–defective) [37,38]and E236Q (Walker B catalytic glutamate; predicted ATP-hydrolysis–defective) [37–39], and generated the K173A/E236Q double mutant (DM) (Figure 4F). Endogenous RIG-I and melanoma differentiation-associated protein 5 (MDA5) were detected after overexpressing empty plasmid, WT TOR3A, and double-mutant TOR3A in HeLa cells. RIG-I and MDA5 are structurally related RLR members [40]. Results showed that both WT and mutant TOR3A reduced the amount of endogenous RIG-I but not MDA5 (Figure 4G), indicating that TOR3A reduces RIG-I protein levels independent of its ATPase activity and specifically targets RIG-I. Co-IP and Western blot analyses revealed interactions between exogenous TOR3A and RIG-I in HEK293 T cells (Figure 4H) and between endogenous TOR3A and RIG-I in RAW264.7 and THP-1 cells (Figure 4I-J). Furthermore, exogenous TOR3A and RIG-I colocalized in HEK293 T cells (Figure 4 K), whereas TOR3A and RIG-I colocalized in RSV-infected PMs (Figure 4L). In summary, these findings indicate that TOR3A regulates IFN-I pathway through RIG-I.

### TOR3A downregulates RIG-I through the ubiquitin-proteasome pathway

**Figure 5.**
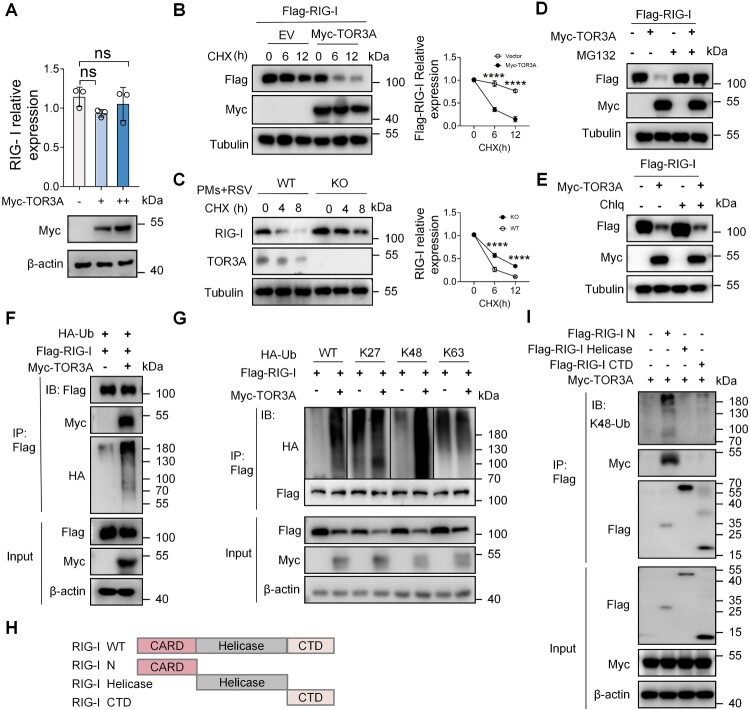
TOR3A downregulates RIG-I in a ubiquitin-proteasome pathway-dependent manner. (**A**) RT-qPCR analysis of endogenous *RIG-I* mRNA levels in HEK293 T cells after 48 h of transfection with increasing amounts of Myc-TOR3A plasmid. (**B**) Western blot analysis of Flag-RIG-I protein stability in HEK293 T cells co-expressing Myc-TOR3A after treatment with cycloheximide (CHX, 50 μg/mL) for indicated times. (**C**) Western blot analysis of RIG-I protein stability in RSV-infected (12 h) WT and TOR3A KO PMs after treatment with cycloheximide (CHX) for the indicated times. (**D**) Western blot analysis of Flag-RIG-I protein levels in HEK293 T cells co-expressing Myc-TOR3A after 6 h treatment with the proteasome inhibitor MG132 (10 μM). (**E**) Western blot analysis of Flag-RIG-I protein levels in HEK293 T cells co-expressing Myc-TOR3A after 6 h treatment with the lysosomal inhibitor chloroquine (CQ). (**F**) Co-IP and Western blot analysis of exogenous RIG-I ubiquitination in HEK293 T cells co-expressing Myc-TOR3A, Flag-RIG-I, and HA-ubiquitin for 48 h. (**G**) HEK293 T cells were co-transfected with Myc-TOR3A, Flag-RIG-I, and various HA-tagged ubiquitin mutants for 48 h, followed by MG132 treatment for 6 h. RIG-I ubiquitination was analyzed by Co-IP and Western blot. (**H**) Schematic diagram of the structural domain of RIG-I. (**I**) Following co-transfection with Myc-TOR3A and Flag-RIG-I domain plasmids for 48 h and MG132 treatment for 6 h, Co-IP and Western blot were performed to assess both the domain-specific interaction with TOR3A and the K48-linked ubiquitination of each RIG-I domain. Statistical significance was determined by student's t-test (B and C), one-way ANOVA followed by Dunett's multiple comparisons test (A). ns: no significance, *P *<* 0.05, **P *<* 0.01, ***P *<* 0.001, ****P *<* *0.0001.*

To determine whether TOR3A regulates RIG-I transcriptionally or post-transcriptionally, TOR3A expression was gradually increased in HEK293 T cells. Results showed TOR3A overexpression did not affect *RIG-I* mRNA levels (Figure 5A), suggesting that TOR3A regulates RIG-I at the post-translational level. Consistent with this result, CHX pulse-chase assay revealed that TOR3A overexpression substantially promoted the degradation of Flag-RIG-I proteins (Figure 5B). The half-life experiment was also conducted in WT and TOR3A KO PMs, and the results showed that the half-life of RIG-I was prolonged in TOR3A KO PMs (Figure 5C). Overall, these results suggest that TOR3A is a regulator of RIG-I protein stability. Protein degradation is mainly mediated by the proteasome and the lysosome system in eukaryotic cells [41,42]. Thus, a proteasome inhibitor MG132 or a lysosomal inhibitor chloroquine Chlq was used to treat TOR3A-overexpressing HEK293 T cells to examine the specific system involved in the regulation of TOR3A on RIG-I degradation. The results showed that MG132, but not Chlq treatment, largely blocked TOR3A-induced RIG-I protein degradation (Figure 5D-E). Collectively, these findings demonstrate that TOR3A induces proteasome-dependent degradation of RIG-I proteins.

Proteasome-dependent protein degradation typically occurs after protein ubiquitination [43]. Therefore, we postulate that TOR3A may regulate the ubiquitination of RIG-I. Consistent with this speculation, Western blot results showed that TOR3A significantly upregulated the polyubiquitination level of exogenous RIG-I (Figure 5F). Furthermore, we analyzed the type(s) of RIG-I ubiquitination mediated by TOR3A. The results showed that TOR3A overexpression markedly promoted K48-linked polyubiquitination of RIG-I, but did not significantly affect other types of RIG-I polyubiquitination (Figure 5G). K48-linked polyubiquitination modification often results in proteasome-mediated degradation. These findings support that TOR3A can induce the ubiquitin-proteasome-dependent degradation of RIG-I.

Next, we mapped the specific domains of RIG-I. Three truncated forms of RIG-I were generated and co-expressed with full-length TOR3A in HEK293 T cells (Figure 5H-I). Co-IP assay results showed that the portion of RIG-I containing caspase activation and recruitment domains (CARDs, amino acids 1–200) interacts with TOR3A, whereas the helicase and carboxyl-terminal domains (CTDs) of RIG-I were not involved in this interaction (Figure 5I). Additionally, TOR3A induced endogenous K48-linked ubiquitination modification of the N-terminal CARD domain of RIG-I (Figure 5I). In summary, these results suggest that TOR3A mediates the degradation of RIG-I through the K48 linked ubiquitin proteasome pathway of the N-terminal CARD domain of RIG-I.

### TOR3A-mediated RIG-I degradation depends on the E3 ubiquitin ligase STUB1

Considering that TOR3A lacks E3 ubiquitin ligase activity, we speculated that an E3 ubiquitin ligase may be involved in TOR3A-mediated K48-linked polyubiquitination and proteasomal degradation of RIG-I. Consequently, by mass spectrometry analysis of the potential TOR3A-interacting proteins, we noticed an E3 ubiquitin ligase STUB1, which displayed many more identified peptides in the TOR3A-overexpressing group than in the vector control group (Figure 6A). STUB1 was finally selected as a candidate by comparing the mass spectrometry analysis results with the predicted results of the Ubibrowser E3 database (Figure 6B).

**← Figure 6.**
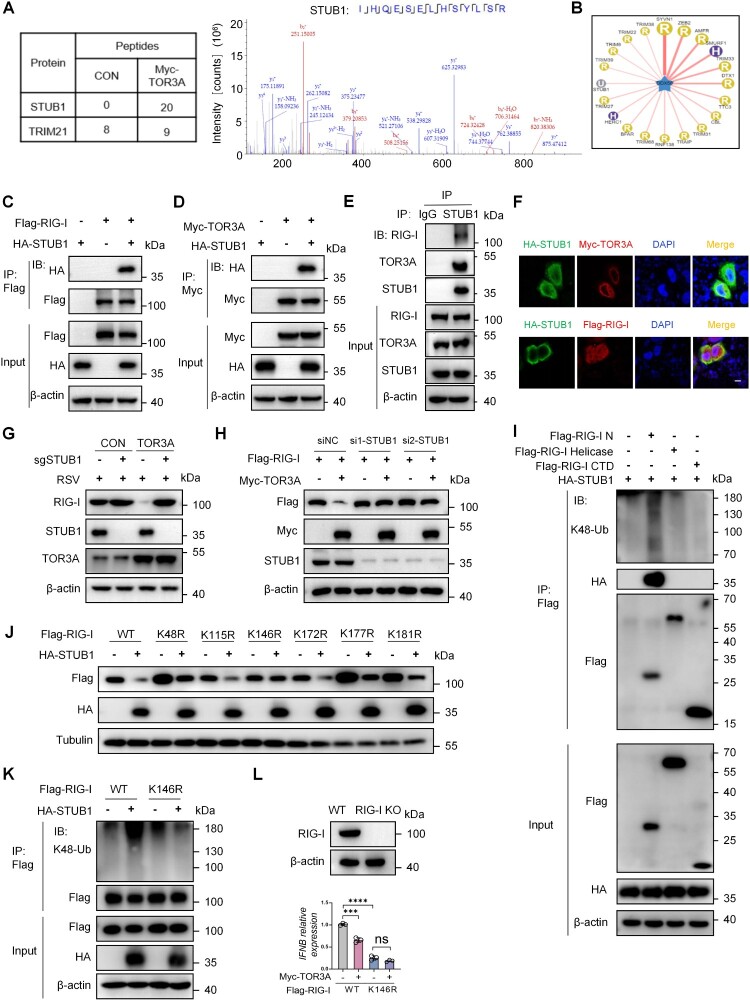
TOR3A targets RIG-I for degradation via the E3 ubiquitin ligase STUB1. (**A**) Mass spectrometry analysis of Myc-TOR3A-interacting proteins co-immunoprecipitated from HEK293 T cells identifies the E3 ubiquitin ligase STUB1 (n = 2). (**B**) Prediction of potential E3 ubiquitin ligases for RIG-I (DDX58) using the UbiBrowser database. (**C**) Co-IP and Western blot analysis of the interaction between Flag-RIG-I and HA-STUB1 in HEK293 T cells 8 h after co-transfection. (**D**) Co-immunoprecipitation (Co-IP) and Western blot analysis of the interaction between Myc-TOR3A and HA-STUB1 in HEK293 T cells after 48 h of co-transfection. (**E**) Co-IP of STUB1 in HeLa cells, followed by Western blot analysis to detect co-precipitated RIG-I and TOR3A. (**F**) Confocal microscopy analysis of the co-localization among HA-STUB1, Myc-TOR3A, and Flag-RIG-I in HeLa cells after 48 h of transfection. Scale bar, 10 μm. (**G**) Western blot analysis of RIG-I protein levels in RSV-infected (12 h) RAW264.7 cells with STUB1 knockout, with or without TOR3A overexpression. (**H**) Western blot analysis of exogenous Flag-RIG-I levels in HEK293 T cells after sequential transfection: first with STUB1-targeting siRNAs (si1, si2) for 24 h, followed by co-transfection with Myc-TOR3A and Flag-RIG-I plasmids for 48 h. (**I**) HEK293 T cells were co-transfected with HA-STUB1 and various Flag-tagged RIG-I domain plasmids for 48 h. Cell lysates were then subjected to co-immunoprecipitation (Co-IP) to assess both the interaction between STUB1 and each RIG-I domain, and the K48-linked ubiquitination levels on these domains. (**J**) Western blot analysis of Flag-RIG-I protein levels in HEK293 T cells co-expressing HA-STUB1 with either wild-type (WT) or site-mutated Flag-RIG-I plasmids for 48 h. (**K**) Co-IP analysis of K48-linked ubiquitination on wild-type (WT) or K146R mutant Flag-RIG-I in HEK293 T cells co-expressing HA-STUB1, following MG132 treatment. (**L**) RT-qPCR analysis of *IFNB* mRNA in RIG-I KO HEK293 T cells, reconstituted with wild-type or K146R mutant RIG-I and Myc-TOR3A, after RSV infection. Data are representative of three independent experiments and presented as mean ± SD. Statistical significance was determined by Student's t-test (L). ns: no significance, *P *<* 0.05, **P *<* 0.01, ***P *<* 0.001, ****P *<* *0.0001.*

Next, we verified the interaction between TOR3A and STUB1 and examined whether STUB1 could also interact with RIG-I. HEK293 T cells were cotransfected with HA-STUB1 and Flag- RIG-I, or Myc-TOR3A and subjected to immunoprecipitation and immunoblot analysis with anti-Flag or anti-Myc antibodies. The results showed that STUB1 co-immunoprecipitated with both RIG-I and TOR3A (Figure 6C-D). Moreover, an endogenous interaction was found between STUB1, RIG-I or TOR3A (Figure 6E). Confocal microscopy assay corroborated the colocalization between endogenous STUB1 and TOR3A or RIG-I (Figure 6F). Moreover, we examined whether TOR3A-mediated RIG-I degradation depends on STUB1. By using the Cas9 technology, we deleted the STUB1 gene in RAW264.7 cells that were overexpressing TOR3A. Once these cells were infected with RSV, the overexpression of TOR3A was unable to inhibit the expression of RIG-I (Figure 6G). Subsequently, in HEK293 T cells, we found that STUB1 knockdown significantly inhibited Flag-RIG-I degradation induced by TOR3A overexpression, as also shown in (Figure 6H). Overall, this data suggests that TOR3A-mediated RIG-I degradation depends on STUB1. Next, we explored the form and sites of STUB1-mediated RIG-I ubiquitination. It was found that STUB1 induced the K48-linked ubiquitination modification of the RIG-I N-terminal CARD domain, consistent with the effect of TOR3A on RIG-I ubiquitination (Figure 6I). We first surveyed reported or predicted ubiquitination-prone lysine residues in the N-terminal CARD domain of RIG-I using the PhosphoSitePlus database and relevant literature [44], and selected candidate sites for validation. We then mutated six lysine residues in the RIG-I CARD domain (K48, K115, K146, K172, K177, and K181) to arginine. Results showed that STUB1 downregulated five RIG-I mutants: RIG-I-K48R, RIG-I-K115R, RIG-I-K172R, RIG-I-K177R, and RIG-I-K181R-to a similar extent as RIG-I-WT (Figure 6J), whereas the K146R mutation partially attenuated STUB1-mediated downregulation of RIG-I protein levels, indicating that K146 contributes to STUB1-dependent regulation (Figure 6J). Consistently, the K146R mutation markedly reduced STUB1-mediated ubiquitination of RIG-I (Figure 6 K). Importantly, secondary mass spectrometry further corroborated the presence of ubiquitination at RIG-I K146 (Fig. S5), providing direct biochemical evidence for modification at this residue. Collectively, these data identify K146 as a major STUB1-responsive ubiquitination site on RIG-I. Meanwhile, WT and K146R RIG-I plasmids were transfected into RIG-I KO HEK293 T cells to further investigate the role of the K146R mutation in RIG-I. The results showed that TOR3A suppressed *IFNB* production in RIG-I KO cells complemented with WT-RIG-I after RSV infection. However, RSV did not stimulate *IFNB* production following K146R-RIG-I (K146 is located at the N-terminus of RIG-I) complementation (Figure 6L). This is because TOR3A lost its target for regulating RIG-I and thus failed to exert any regulatory effect. Taken together, Together, these results show STUB1 binds TOR3A and RIG-I to mediate K48-linked ubiquitination and degradation of RIG-I at K146.

### TOR3A promotes STUB1-RIG-I interaction, leading to increased ubiquitination and degradation of RIG-I

To elucidate the role of TOR3A in STUB1-mediated RIG-I degradation, HEK293 T cells were co-transfected with Flag-RIG-I, HA-STUB1 and Myc-TOR3A. The results showed that TOR3A facilitated STUB1-mediated RIG-I degradation (Figure 7A). Further Co-IP experiments revealed that the interaction between STUB1 and TOR3A was notably augmented after TOR3A overexpression in HEK293 T cells (Figure 7B). Moreover, Co-IP results indicated that the interaction between STUB1 and RIG-I increased with the gradient overexpression of TOR3A; the K48-linked ubiquitination modification of RIG-I was also significantly enhanced (Figure 7C). As anticipated, CRISPR-Cas9-mediated TOR3A KO reduced STUB1-RIG-I interaction and K48 ubiquitination of RIG-I in HEK293 T cells (Figure 7D-E). To clarify how STUB1 regulates RIG-I protein levels when TOR3A is knocked out, we overexpressed STUB1 in TOR3A KO cells. The results showed that knocking out TOR3A could significantly prevent STUB1-induced RIG-I degradation (Figure 7F). Taken together, these data suggest that TOR3A promotes STUB1-RIG-I interaction, which subsequently results in enhanced ubiquitination and degradation of RIG-I. Moreover, the STUB1-induced degradation of RIG-I depends on the presence of TOR3A.

**← Figure 7.**
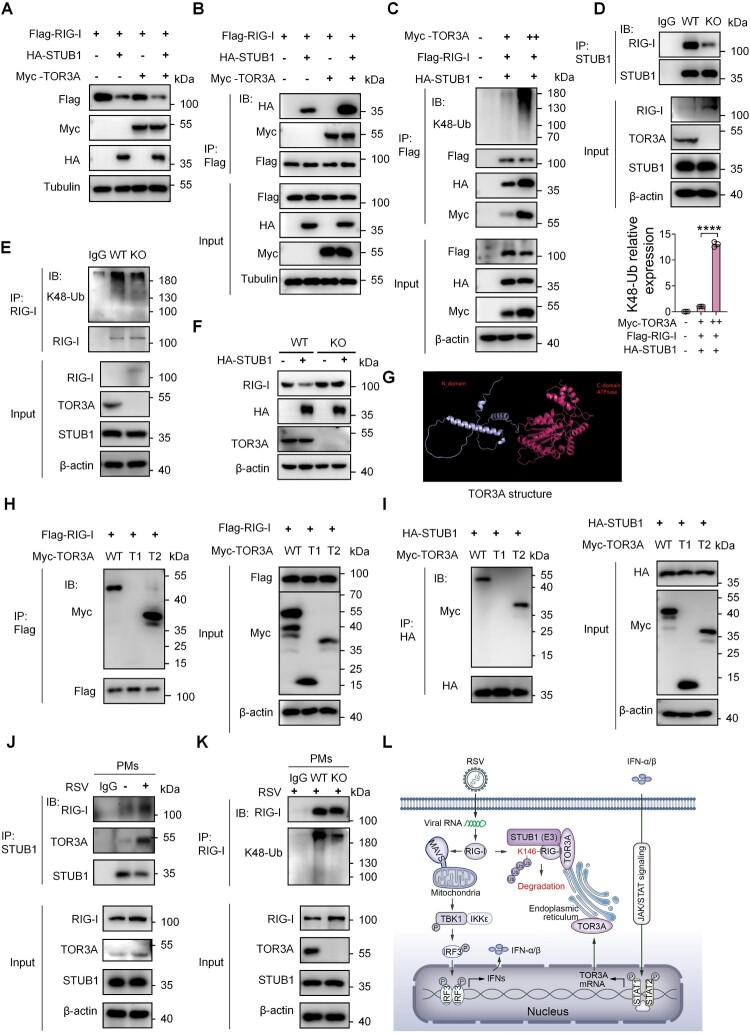
TOR3A facilitates the E3 ligase STUB1 in ubiquitinating RIG-I. (**A**) Western blot analysis of Flag-RIG-I protein levels in HEK293 T cells co-expressing HA-STUB1 and Myc-TOR3A for 48 h. (**B**) Co-IP and Western blot analysis of the interaction between HA-STUB1 and Flag-RIG-I in HEK293 T cells co-expressing Myc-TOR3A for 48 h. (**C**) Co-IP and Western blot analysis of K48-linked ubiquitination on Flag-RIG-I in HEK293 T cells co-expressing HA-STUB1 and Myc-TOR3A. (**D**) Western blot analysis of RIG-I, TOR3A, and STUB1 protein levels in wild-type versus TOR3A-knockout HEK293 T cells. Co-IP and Western blot analysis of the STUB1-RIG-I interaction in the same cell lines. (**E**) Co-IP and Western blot analysis of K48-linked polyubiquitination of RIG-I in wild-type versus TOR3A-knockout HEK293 T cells. (**F**) Western blot analysis of RIG-I protein levels in wild-type and TOR3A-knockout HEK293 T cells after 48 h transfection with the HA-STUB1 plasmid. (**G**) Structural model of TOR3A generated using PyMOL software. (**H**) Co-IP and Western blot analysis of the interaction between Flag-RIG-I and Myc-TOR3A domains in HEK293 T cells. (**I**) Co-IP and Western blot analysis of the interaction between HA-STUB1 and Myc-TOR3A domains in HEK293 T cells after MG132 treatment. (**J**) Western blot analysis of TOR3A, RIG-I, and STUB1 expression, and Co-IP/Western blot analysis of their ternary complex formation, in primary macrophages (PMs) at 12 h post-RSV infection. (**M**) Analysis of RIG-I ubiquitination by immunoprecipitation in RSV-infected (12 h) wild-type versus TOR3A-knockout PMs. (**L**) A model illustrating how TOR3A regulates innate immune signalling against RSV infection. Data are representative of three independent experiments and presented as mean ± SD. Statistical significance was determined by Student's t-test (C). ns: no significance, *P *<* 0.05, **P *<* 0.01, ***P *<* 0.001, ****P *<* *0.0001.*

Next, to determine the specific domain in TOR3A involved in the binding of RIG-I and STUB1, we queried the TOR3A structure predicted by Alphafold in the UniProt database and imported the TOR3A structure into the PyMol software for visualization. We found that H-TOR3A was divided into an N-terminal domain T1 (1–116 aa, 13.39 kDa) and a C-terminal containing ATPase Domain T2 (117–397 aa, 32.83 kDa) (Figure 7G). Co-IP results showed that the TOR3A truncated form containing the T2 domain mediated interactions between TOR3A and RIG-I, as well as TOR3A and STUB1 (Figure 7H-I). Furthermore, we sought to elucidate whether TOR3A upregulation during RSV infection affects the protein level of STUB1 and whether RSV infection influences the interaction between STUB1, RIG-I and TOR3A. Consequently, primary PMs were infected with RSV. Western blot results showed that TOR3A and RIG-I protein levels were significantly increased after RSV infection, while the protein level of STUB1 remained unchanged (Figure 7J). Endogenous Co-IP assay revealed that the interaction between STUB1, RIG-I and TOR3A was significantly enhanced following RSV infection (Figure 7J). Further Co-IP assay also showed that endogenous ubiquitination of RIG-I in PMs of TOR3A KO was significantly decreased (Figure 7 K). Collectively, these findings suggest elevated TOR3A levels enhance STUB1-RIG-I interaction post-RSV infection, resulting in RIG-I ubiquitination and degradation, which suppresses the IFN-I signalling pathway and promotes RSV replication. Together, our findings indicate that RSV infection induced macrophages to produce IFN-I, which stimulated the transcription factor STAT1 to upregulate TOR3A expression in macrophages. Subsequently, through its C-terminal domain, TOR3A interacted with the N-terminus of RIG-I in the IFN-I pathway and the E3 ubiquitin ligase STUB1, and this interaction activated the STUB1-mediated K48-linked polyubiquitination modification and proteasome degradation at the K146 site of RIG-I. (Figure 7L).

## Discussion

ISGs typically inhibit viral infection by targeting various stages of the viral life cycle [45], though some paradoxically enhance viral replication by suppressing IFN signalling or directly promoting infection [20,46]. As a torsin family member, TOR3A remains relatively understudied [27]. Here we identify TOR3A as a STAT1-responsive negative regulator of antiviral immunity during RSV infection, negatively regulating antiviral immunity by suppressing IFN-β production, potentially serving to modulate immune responses and prevent excessive inflammation. Our study demonstrated that the expression of TOR3A is significantly upregulated in RAW264.7 cells following RSV infection. In line with this, TOR3A was highly expressed in samples from mice and children infected with RSV, suggesting that TOR3A may participate in the host's innate immunity against RSV. As a key transcription factor downstream of the IFN-I signalling pathway, the binding of STAT1 to the TOR3A promoter may directly initiate the transcription process associated with the TOR3A gene. This discovery provides important insights into the role of TOR3A in immune responses. In the future, investigating the specific sites and regulatory modes of the STAT1 binding to the TOR3A promoter may uncover deeper regulatory mechanisms of TOR3A expression and its changes under different physiological and pathological conditions.

Moreover, our results indicate that TOR3A overexpression reduces RIG-I protein levels. RIG-I is a key cytosolic pattern-recognition receptor (PRR) that senses RSV RNA and initiates antiviral signalling, and its activity is tightly regulated to ensure appropriate activation and timely resolution of innate immune responses [47–49]. Upon RNA virus invasion, RIG-I undergoes a notable conformational shift from a self-repressed state to an active form. In this activated state, it sequesters the viral RNA within a central cavity formed by the helicase and C-terminal domains, while simultaneously exposing the N-terminal CARD domain. This exposed domain interacts with downstream MAVS to initiate antiviral signalling [50]. To date, several E3 ubiquitin ligases have been implicated in RIG-I degradation, including RNF125 [51], c-Cbl [52], and STUB1 [53,54]. Studies have also identified co-factors that facilitate the deubiquitinase (DUB) or E3 ubiquitin ligase leading to the negative regulation of RIG-I. Among them, SDC4 was identified to interact with RIG-I and CYLD, reducing the K63-linked ubiquitination of RIG-I to prevent excessive IFN-I synthesis [55]. In our study, we found that TOR3A was a novel negative regulator of RIG-I, comprising an N-terminal and C-terminal domain containing incomplete ATPase. However, it lacks the E3 ubiquitin ligase activity, but can enhance the degradation of RIG-I by STUB1 owing to its ubiquitin-proteasome pathway.

STUB1 is an important E3 ubiquitin ligase known to regulate various cellular biology processes, such as protein quality control, signal transduction, and heat shock response etc. by interacting with specific target proteins and attaching ubiquitin chains [56]. Currently, the majority of studies have focused on the ubiquitination of its target substrates [57]. Studies have identified several targets and downstream functions, such as regulation of necroptosis via RIPK1 [58] and modulation of T cells function through FoxP3 [59]. Zhao et al. reported that STUB1 targets the degradation of RIG-I [53], but the role of STUB1 and TOR3A in RIG-I under RSV infection has not been studied. Some studies have reported that RIG-I is redistributed to the membrane in a perinuclear pattern and participates in antiviral signal transduction [60]. TOR3A is mainly located in the endoplasmic reticulum membrane and nuclear membrane, whereas STUB1 is mainly located in the cytoplasm, endoplasmic reticulum and nucleus, which enables the spatial identification of RIG-I redistribution after viral infection. In this study, the results indicated that the expression level of the STUB1 protein was not altered following RSV infection, but its binding to RIG-I and TOR3A increased significantly. Although the total amount of STUB1 did not change, more TOR3A bound to STUB1 after RSV infection and promoted its ubiquitination modification to degrade RIG-I, inhibit the IFN-I pathway, stimulate RSV replication and immune escape.

Clinical sample and public transcriptomic data showed that *TOR3A* mRNA expression in PBMCs of patients with RSV infection was significantly increased and positively correlated with disease severity and viral load, suggesting an association with the host antiviral response. ROC analysis showed that *TOR3A* expression showed moderate discriminative performance in this cohort (AUC = 0.719) for differentiating RSV-associated bronchiolitis, and analysis of the GSE164015 dataset similarly supported elevated *TOR3A* expression in hospitalized RSV patients with comparable discrimination (AUC = 0.7587). PBMC *TOR3A* levels may be influenced by potential confounding factors, including changes in leukocyte composition during infection and variability in sampling time relative to symptom onset and treatment. Therefore, the clinical utility of *TOR3A* as a biomarker requires further validation in larger, prospective cohorts, ideally alongside established diagnostic measures such as viral antigen detection and nucleic acid quantification [61,62].

In summary, our study demonstrates that TOR3A inhibits the RIG-I-mediated anti-RSV immune response, laying the foundation for further comprehensive investigations into the body's anti-RSV innate immune mechanism, and identification of new targets for RSV infection-related diseases (bronchiolitis and pneumonia). Together with the consistent effects observed in VSV and SeV models, these data indicate that TOR3A may act in a non-pathogen-specific manner to modulate innate antiviral responses, highlighting TOR3A as a candidate host factor for broad-spectrum antiviral strategies.

## Materials and methods

### Mice

TOR3A knockout mice and genotyping. Wild-type (WT) and knockout (KO) alleles were identified by PCR with allele-specific primers (WT: 5′-CCTCACCTGCCCTGATTTCTC-3′/5′-TTCTCACCAGTCGCTTCTCCATA-3′; KO: 5′-CCCTGCTCAGACGGTTTCAT-3′/5′-GGGTAGGGAGGGCAGAGTAA-3′). The C57BL/6 TOR3A KO line was generated by Biomodel Organism Science & Technology Development Co., Ltd. (Shanghai, China) using CRISPR/Cas9. Guide RNAs targeting Tor3a were designed in silico and produced by in-vitro transcription; sequences are listed in Table S2. Mice on a C57BL/6 background were kept under specific pathogen-free (SPF) conditions in the Soochow University animal facility.

### Human peripheral blood samples

PBMCs from children in the RSV-infected and control groups were isolated from residual clinical blood samples originally collected for routine diagnostic purposes by the Medical Laboratory Department of the Children's Hospital of Soochow University. The study was approved by the Ethics Committee of the Children’s Hospital of Soochow University, and written informed consent was obtained from at least one legal guardian before enrolment. Between 15 November 2022 and 18 April 2023, we enrolled 70 inpatients with bronchiolitis at the Children’s Hospital of Soochow University and recruited an age-matched elective-surgery control group (n = 40). Diagnosis and severity grading followed the 2024 Clinical Practice Guideline for bronchiolitis. Respiratory syncytial virus (RSV) infection was confirmed by antigen testing of nasopharyngeal aspirates. Additional microbiologic evaluations were performed to exclude concomitant respiratory infections, and no other pathogens were detected following the approach described by Song et al. [63]_._ Peripheral blood was collected at admission, and all patient data were anonymized prior to analysis.

### Cell culture and reagents

RAW264.7, HEK293 T, HeLa, and Hep-2 cells (ATCC) were cultured in DMEM (HyClone) containing 10% FBS (Biological Industries), penicillin (100 U/mL; NCM Biotech), and streptomycin (100 μg/mL; NCM Biotech) at 37 °C in a humidified 5% CO₂ atmosphere. PMs were isolated from 6–8-week-old C57BL/6J mice following intraperitoneal injection of a 4% thioglycollate solution. THP-1 cells (ATCC) and PMs were cultured in RPMI 1640 (HyClone) containing 10% FBS (Biological Industries), penicillin (100 U/mL; NCM Biotech), and streptomycin (100 μg/mL; NCM Biotech) at 37 °C in a humidified 5% CO₂ atmosphere. Innate immune stimuli included poly(I:C) (Merck), 5′-ppp hairpin RNA, and the STING agonist DMXAA (both from InvivoGen); recombinant mouse IFN-β and IFN-λ2 was from R&D Systems. Cycloheximide (Sigma-Aldrich) and additional laboratory reagents were used as indicated. All cell lines were supplier-authenticated and routinely verified mycoplasma-negative prior to experimentation.

### Establishment of STUB1 KO RAW264.7 cell line through electroporation-based CRISPR/Cas9 gene editing technology

Three CRISPR gRNAs targeting the mouse STUB1 gene were designed using the Genscript website. These sequences are presented in Table S3. Then, sgRNAs, GenCRISPR™ Cas9 v1.2 protein (Genscript) and electroporation solution (Celetrix) were mixed and left to stand at room temperature for 10 min. RAW264.7 cells to be edited were washed with PBS and then centrifuged. Then the cell pellet was mixed with the electroporation mixture containing sgRNAs and Cas9 protein and added to the electroporation tube (Celetrix). The electroporation tube was subsequently placed in an electroporator (Celetrix) and electroporated at 530 V (voltage). The construction method of TOR3A KO HEK293 T cell line is the same as that of RAW264.7 cell line. The sequences are shown in Table S4.

### Transfection

HA ubiquitin (HA-Ub), HA-Ub-K27 (all lysines on the ubiquitin gene are mutated to arginines except K27), HA-Ub-K48, and HA-Ub-K63 plasmids were gifts from Professor Hui Zheng (University of Electronic Science and Technology, China). pLV-Flag-RIG-I N, pLV-Flag-RIG-I Helicase and pLV-Flag-RIG-I CTD plasmids were gifts from Professor Fang Fang Zhou (Soochow University, China). Other plasmids were purchased from Changsha Ruiying Biotechnology. The pLL3.7-sh-m-TOR3A and pLL3.7-sh-H-TOR3A were constructed into pLL3.7 plasmid. All transient transfections were carried out using Lipofectamine 3000 (Thermo Fisher Scientific) according to the manufacturer’s instructions. Control siRNA (catalog no. 160818), siTOR3A and siSTUB1 siRNAs were synthesized by RiboBio Co.(Guangzhou, China). siRNAs (25 pmol) were used to transfect HEK293 T cells in 12-well plates using GP-transfect-Mate (GenePharma) according to the manufacturer's instructions.

### Virus preparation, titration, and infection

RSV (L19 strain) was provided by Prof. Chunsheng Dong (Soochow University, China) and expanded in Hep-2 cells. VSV-GFP and SeV were gifts from Prof. Hui Zheng (University of Electronic Science and Technology, China). VSV was a gift from Dr. Fangfang Zhou (Soochow University, China). Infectious titres were determined by plaque assay using standard procedures as described previously [9], and viruses were stored at –80 °C until use. For in-vitro infection, RAW264.7 cells or primary PMs were exposed in 2% medium to RSV (MOI = 1), VSV (MOI = 0.1), or SeV (100 HAU/mL) for 2 h to allow entry, followed by replacement with complete medium for downstream analyses (RT-qPCR/Western blot). For in-vivo studies, 6–8-week-old WT or TOR3A-KO mice were anesthetized with isoflurane and inoculated intranasally with 1 × 10^6^ PFU RSV in 2% DMEM or PBS; lungs were collected at 3 dpi for RT-qPCR, Western blot, plaque assay, and histology. WT or TOR3A-KO mice received 4 × 10^8^ PFU VSV per mouse (intraperitoneal); viral loads in lungs, kidneys, spleens, and livers were quantified by RT-qPCR or Western blot, and survival was monitored thereafter. All procedures involving live virus were conducted in BSL-2 laboratories.

### RNA isolation, RT-qPCR

Total RNA was purified from cultured cells and mouse tissues using RNA-easy Isolation Reagent (Vazyme). First-strand cDNA was synthesized with the HiScript III 1st Strand cDNA Synthesis Kit (YEASEN). Quantification was performed by SYBR Green RT-qPCR (YEASEN) on an Applied Biosystems platform with gene-specific primers. The relative expression of the target genes was normalized to 18S mRNA. The sequences for primers are listed in Table S5.

### Western blot and immunoprecipitation

Whole-cell lysates were prepared and clarified by centrifugation (12,000 rpm, 20 min). For co-immunoprecipitation, clarified lysates were incubated with the indicated antibodies together with Protein G-Agarose beads (Roche) for endogenous Co-IP. For epitope-tag Co-IP, lysates were immunoprecipitated using anti-FLAG magnetic beads (B26101, Selleck) or anti-Myc magnetic beads (B26301, Selleck) as indicated. Immunoprecipitates were washed and then subjected to SDS-PAGE and immunoblotting with the indicated antibodies. Signals were developed on a Tanon 5200 chemiluminescence system (Tanon Science & Technology, Shanghai, China) using Western ECL substrate (FDbio Science, Hangzhou, China). Antibodies and working dilutions were: TOR3A (10647-1-AP, Proteintech), RIG-I (67556-1-Ig, Proteintech), p-TBK1 (3504S, CST), TBK1 (24930S, CST), p-IRF3 (catalog as indicated, CST), IRF3 (catalog as indicated, CST), HA (T0008, Affinity Biosciences), Myc (AF6055, Affinity), FLAG (T0003, CST), RSV-M2-1 (ab1874, Abcam), STUB1 (A11751, ABclonal), Ubiquitin (sc-8017, Santa Cruz), β-actin (T0022, Affinity), GAPDH (FD0063, FDbio Science), and Tubulin (MAN1002, MESGEN).

### Confocal microscopy assay

Cells were fixed (4% paraformaldehyde, 30 min, RT), permeabilized (0.2% Triton X-100, 10 min), and blocked (3% BSA in PBS, 1 h). Then, the cells were stained with rabbit polyclonal antibody to RIG-I, mouse polyclonal antibody to TOR3A, followed by staining with either 488 goat anti-rabbit IgG (1:500, Southern Biotech, 4050–30) or 647 goat anti-mouse IgG (1:500, Invitrogen, A21235). Cell nuclei were stained with DAPI, and the fluorescent images were captured with the Nikon confocal microscope.

### Luciferase reporter assay

HEK293 cells were transfected with a TOR3A promoter-firefly construct together with pRL-TK and either a STAT1 expression plasmid or empty vector. At 36 h post-transfection, reporter output was quantified with the dual-luciferase kit (YEASEN, 11402ES60) and expressed as Firefly/Renilla according to the manufacturer’s instructions.

### Mass spectrometry

Proteomic sample preparation, LC–MS/MS acquisition, and data processing were performed as previously described in our recent study [64]. The mass spectrometry datasets have been deposited in the iProX repository (http://www.iprox.cn) under the accession numbers IPX0015410000, IPX0015414000, and IPX0015415000.

### Statistical analysis

Statistical analyses and ROC cure were performed in GraphPad Prism 10, consistent with prior practice in similar studies [65]. Data are presented as mean ± standard deviation (SD) from three or more independent experiments unless otherwise noted. Two-sided unpaired Student's t-test was used for two groups comparison, one-way analysis of variance (ANOVA) with post Tukey's multiple comparisons test, and two-way ANOVA with post-Sidak's multiple comparisons test were used for multi-group comparison. Categorical data were expressed as counts and percentages, and the χ2 test was used for group comparisons. Correlations were tested using Spearman’s ρ. *P* *<* *0.05* was considered statistically significant. *ns: no significance,* * *P* < 0.05; ** *P* < 0.01; *** *P* < 0.001; **** *P* < 0.0001.

## Supplementary Material

Supplementary_Material-clean.doc

FigS3.jpg

FigS4.jpg

FigS2.jpg

FigS1.jpg

FigS5.jpg
