# Supplementary material for: TOR3A represses type I interferon production and limits viral clearance during respiratory syncytial virus infection
Source: Emerg Microbes Infect. 2026 Feb 25;15(1):2637961. doi: 10.1080/22221751.2026.2637961 (PMC12990277; doi:10.1080/22221751.2026.2637961)
Supplement: Supplementary_Material-clean.doc [file TEMI_A_2637961_SM9109.doc]

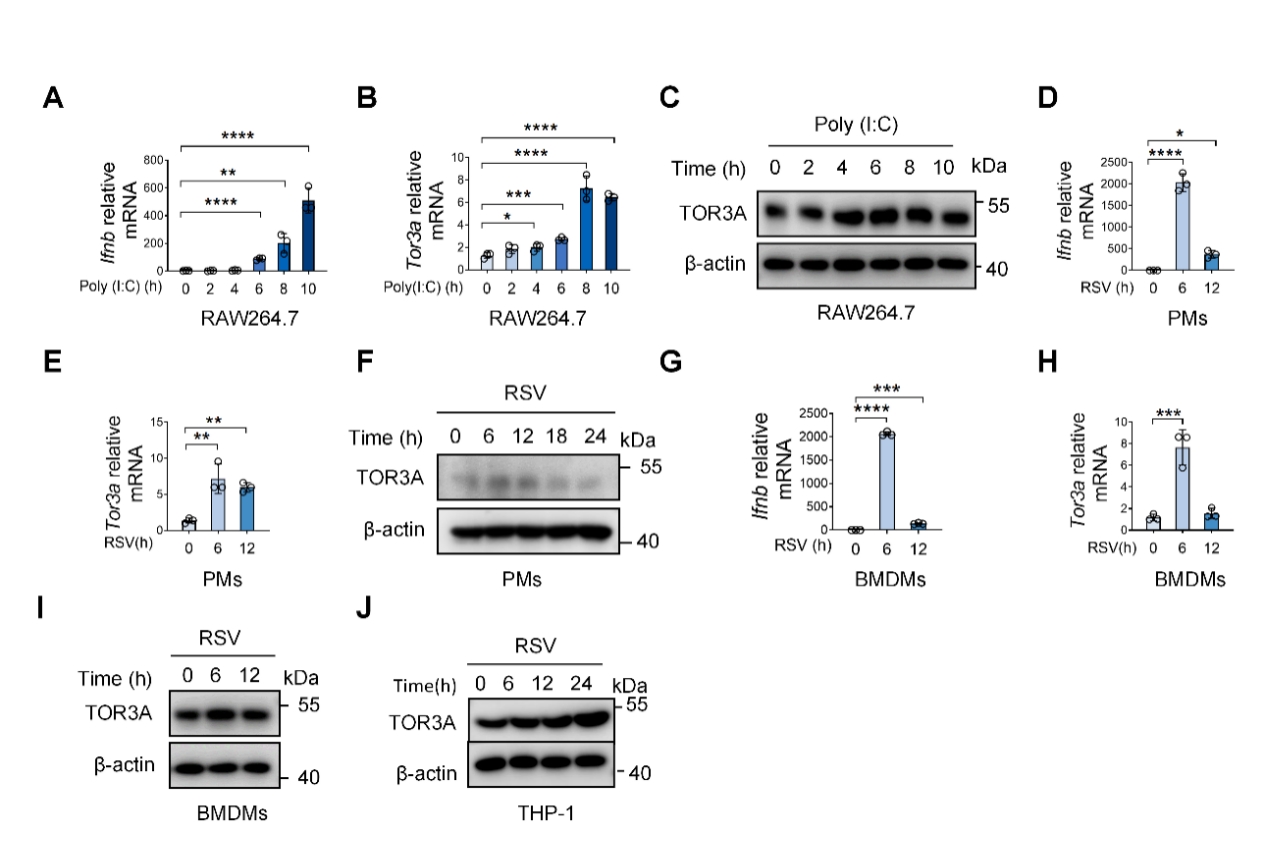


**Figure S1. RSV infection induces TOR3A expression in macrophages.** (**A-B**) RT-qPCR analysis of *Ifnb* and *Tor3a* mRNA levels in RAW264.7 cells at indicated time points after transfection with Poly(I:C) (1 μg/mL). (**C**) Western blot analysis of TOR3A protein expression in RAW264.7 cells after transfection with Poly(I:C) (1 μg/mL). (**D-E**) RT-qPCR analysis of *Ifnb* and *Tor3a* mRNA levels in PMs at indicated time points after RSV infection. (**F**) Western blot analysis of TOR3A protein expression in PMs after RSV infection. (**G-H**) RT-qPCR analysis of *Ifnb* and *Tor3a* mRNA levels in BMDMs at indicated time points after RSV infection. (**I**) Western blot analysis of TOR3A protein expression in BMDMs after RSV infection. (**J**) Western blot analysis of TOR3A protein expression in THP-1 cells after RSV infection. Data are representative of three independent experiments and presented as mean ± SD. Statistical significance was determined by one-way ANOVA followed by Dunnett's multiple comparisons test (A, B, D, E, G and H). *ns:* *no significance, *P<0.05, **P<0.01, ***P<0.001, ****P<0.0001.*


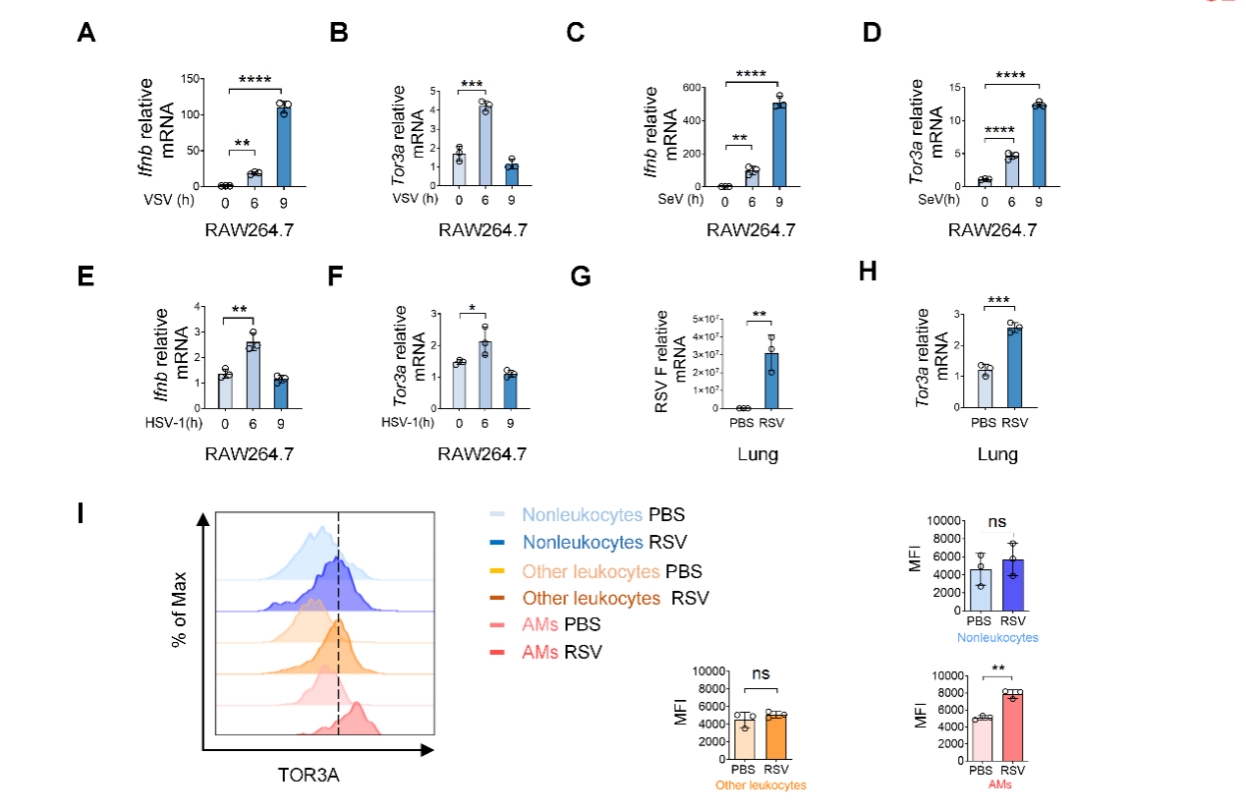


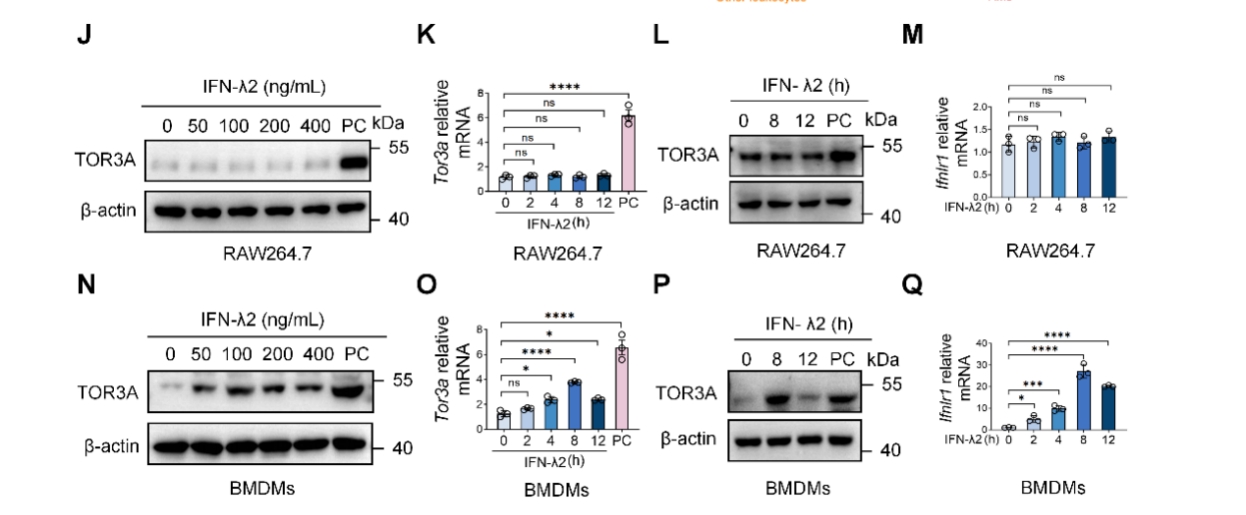


**Figure S2. Both RNA viruses and DNA viruses can upregulate the expression of TOR3A.** (**A-B)** RT-qPCR analysis of *Ifnb* and *Tor3a* mRNA levels in RAW264.7 cells after VSV infection. (**C-D**) RT-qPCR analysis of *Ifnb* and *Tor3a* mRNA levels in RAW264.7 cells after SeV infection.(**E-F**) RT-qPCR analysis of *Ifnb* and *Tor3a* mRNA levels in RAW264.7 cells after HSV-1 infection. (**G-H**) RT-qPCR analysis of RSV-F and *Tor3a* mRNA levels in lung tissues of C57BL/6 mice at 3 days post-intranasal RSV infection. (**I**) Flow cytometry analysis of TOR3A mean fluorescence intensity (MFI) in specified lung cell subsets from C57BL/6J mice after intranasal RSV infection. AMs refers to a group of CD45^+^, Siglec-F^+^ (CD170^+^), CD11b moderately expressed, CD11c^+^ and CD64^+^ cells. Other leukocytes are CD45 positive and CD170, CD11b, CD11 and CD64 negative cell populations. Nonleukocytes are CD45-negative cells. (**J**) Western blot analysis of TOR3A protein expression in RAW264.7 cells stimulated with indicated doses of mouse IFN-λ2. PC: positive control (mouse IFN-β, 8 h). (**K**) RT-qPCR analysis of *Tor3a* mRNA levels in RAW264.7 cells at indicated time points after stimulation with mouse IFN-λ2 (100 ng/mL). **(L)** Western blot analysis of TOR3A protein levels in RAW264.7 cells at indicated time points after stimulation with mouse IFN-λ2 (100 ng/mL). **(M)** RT-qPCR analysis of *Ifnlr1* mRNA levels in RAW264.7 cells at indicated time points after stimulation with mouse IFN-λ2 (100 ng/mL). **(N)** Western blot analysis of TOR3A protein levels in BMDMs stimulated with indicated doses of mouse IFN-λ2. **(O)** RT-qPCR analysis of *Tor3a* mRNA levels in BMDMs at indicated time points after stimulation with mouse IFN-λ2 (100 ng/mL). **(P)** Western blot analysis of TOR3A protein levels in BMDMs at indicated time points after stimulation with mouse IFN-λ2 (100 ng/mL). **(Q)** RT-qPCR analysis of *Ifnlr1* mRNA levels in BMDMs at indicated time points after stimulation with mouse IFN-λ2.Data are representative of three independent experiments and presented as mean ± SD. Statistical significance was determined by Student's t-test (G, H, and I ), one-way ANOVA followed by Dunett's multiple comparisons test (A, B, C, D, E F, K, M, O and Q). *ns:* *no significance, *P<0.05, **P<0.01, ***P<0.001, ****P<0.0001.*


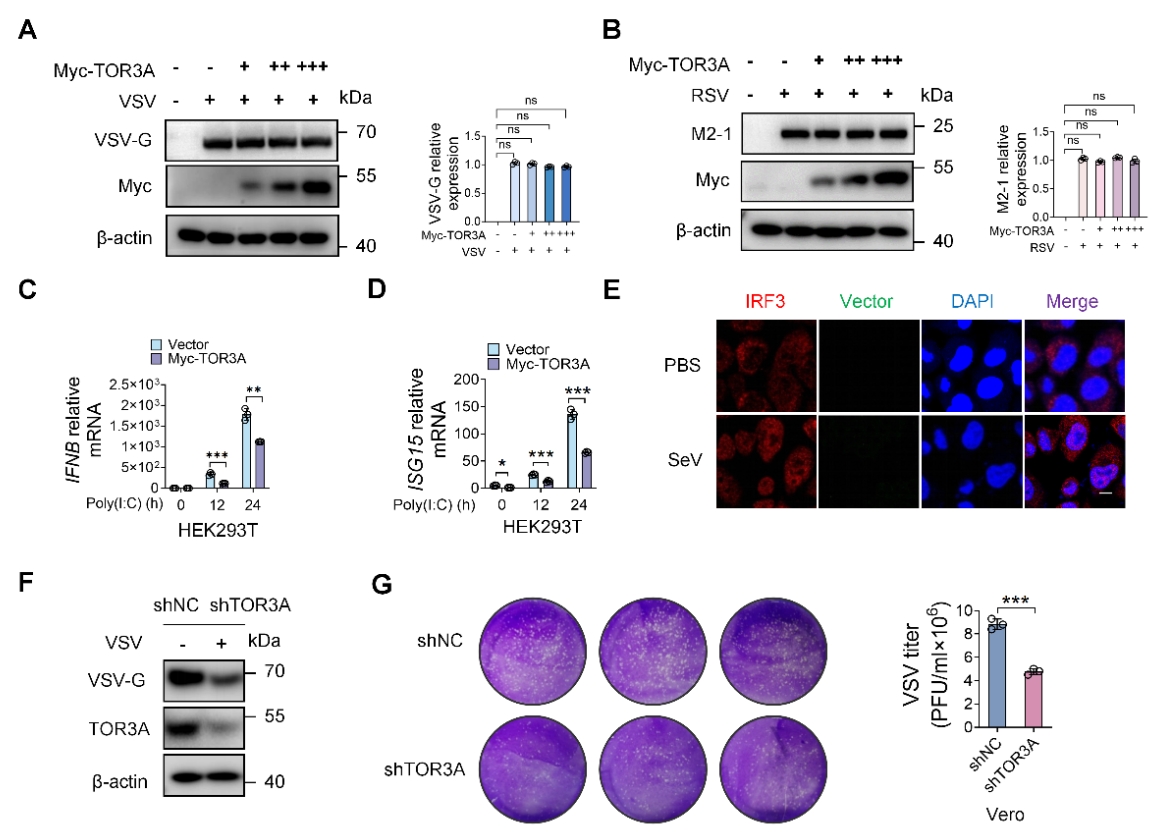


**Figure S3. TOR3A inhibits the production of IFN-β and promotes the replication of RNA viruses.** (**A**) Western blot analysis of VSV-G protein in Vero cells at 12 h post-VSV infection, following 24 h of transfection with Myc-TOR3A. (**B**) Western blot analysis of RSV-M2-1 protein in Vero cells at 12 h post-RSV infection, following 24 h of transfection with Myc-TOR3A. (**C-D**) RT-qPCR analysis of *IFNB* and *ISG15* mRNA levels in HEK293T cells after sequential transfection with Myc-TOR3A (24 h) and stimulation with Poly(I:C) (1 μg/mL). (**E**) After overexpressing empty vector (EV, control) in HeLa cells, the cells were infected with SeV for 12 h and stained with IRF3 (red) and Myc (green) antibodies (scale bar, 10 μm). (**F**) Western blot analysis of VSV-G protein in HEK293T cells at 12 h post-VSV infection, following 36 h of transfection with TOR3A-targeting shRNA. (**G**) Plaque assay of viral titers in supernatants from HEK293T cells at 24 h post-VSV infection, following 36 h of transfection with TOR3A-targeting shRNA. Data are representative of three independent experiments and presented as mean ± SD. Statistical significance was determined by student's t-test (C, D, and F). *ns:* *no significance, *P<0.05, **P<0.01, ***P<0.001, ****P<0.0001.*


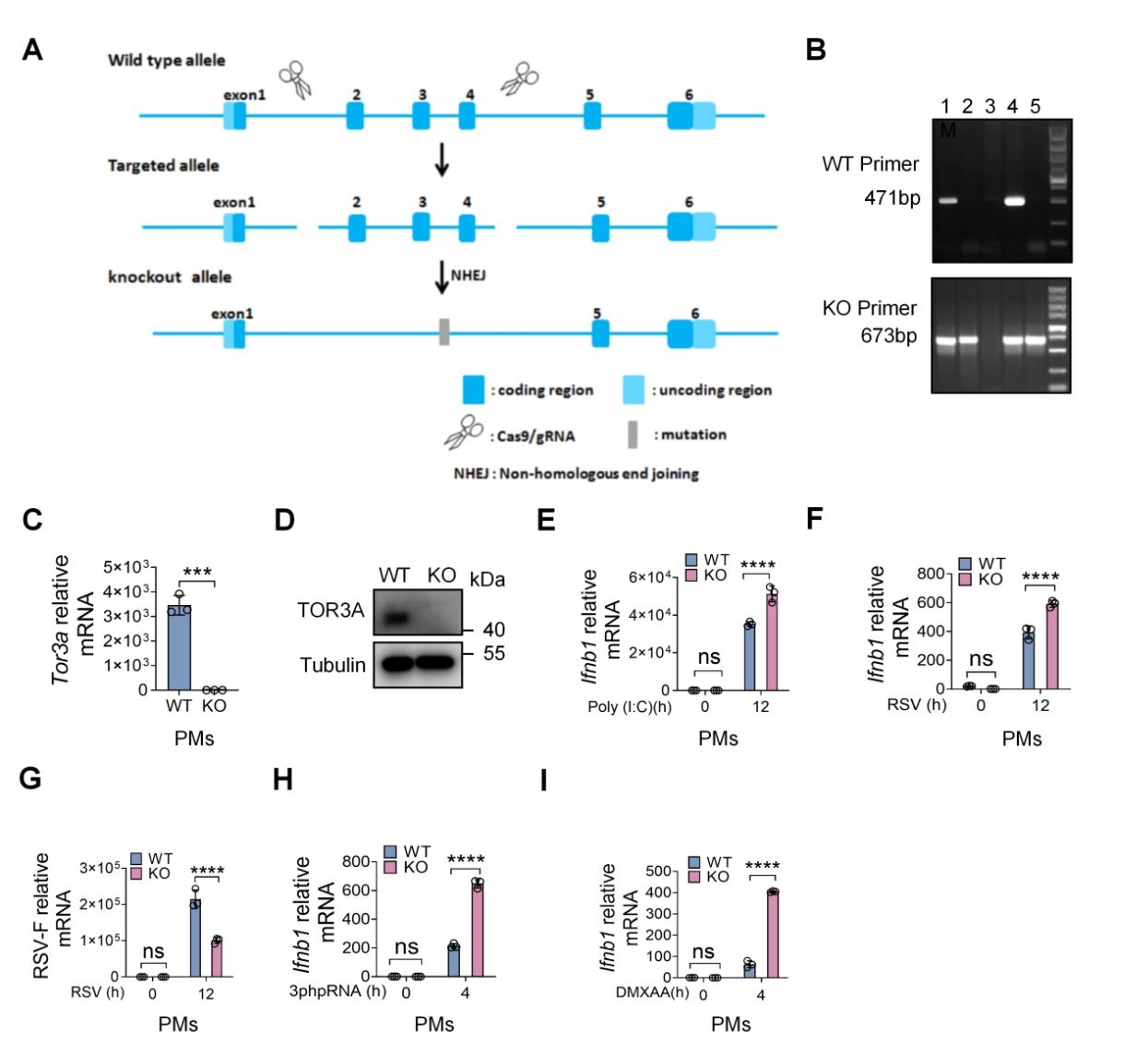


**Figure S4. TOR3A deficiency potentiates anti-RSV viral response.** (**A**) Strategy for the generation of TOR3A-KO mice using CRISPR-Cas9 technology. (**B**) Genotyping of mice by PCR analysis of tail DNA. (**C**) RT-qPCR analysis of *Tor3a* mRNA levels in PMs from WT and TOR3A KO mice. (**D**) Western blot analysis of TOR3A protein levels in PMs from WT and TOR3A KO mice. (**E**) RT-qPCR analysis of *Ifnb* mRNA levels in Poly(I:C) (1 μg/mL)-transfected PMs from WT and TOR3AKO mice. (**F**) RT-qPCR analysis of *Ifnb* mRNA levels in RSV-infected PMs from WT and TOR3A KO mice. (**G**) RT-qPCR analysis of RSV-F mRNA levels in RSV-infected PMs from WT and TOR3A KO mice. (**H**) RT-qPCR analysis of *Ifnb* mRNA levels in PMs from WT and TOR3A KO mice after transfection with 3p-hpRNA (300 ng/mL). (**I**) RT-qPCR analysis of *Ifnb* mRNA levels in PMs from WT and TOR3A KO mice after stimulation with DMXAA (10 μg/mL). Data are representative of three independent experiments and presented as mean ± SD. Statistical significance was determined by Student's t-test (C, E, F, G, H, and I). *ns:* *no significance, *P<0.05, **P<0.01, ***P<0.001, ****P<0.0001.*


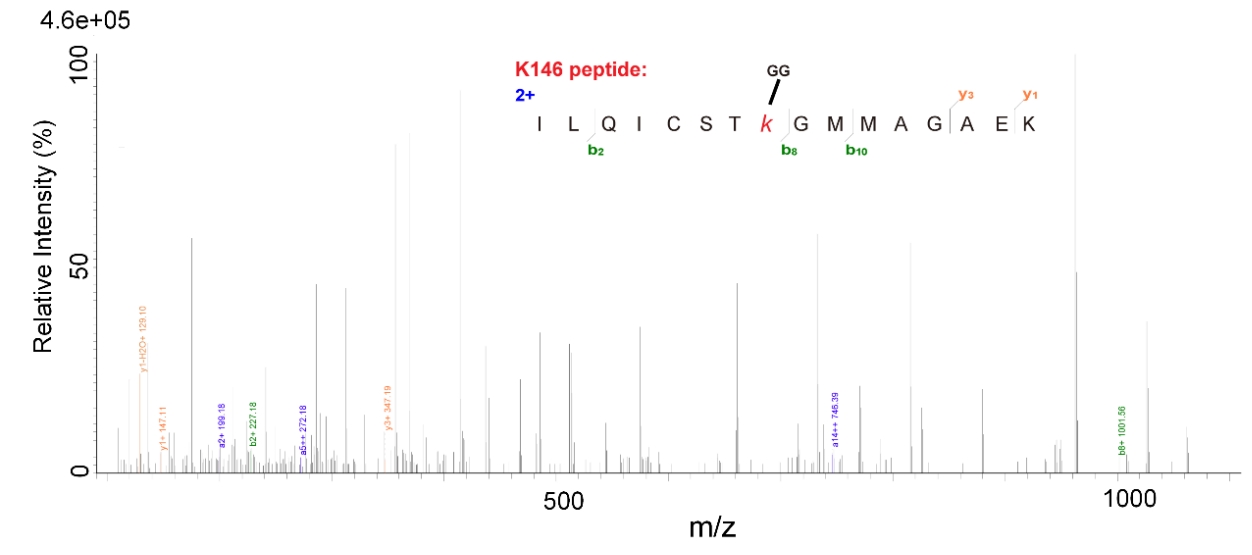
**Figure S5. Identification of K146 as a ubiquitination site on RIG-I.** HEK293T cells were co-transfected with Flag-RIG-I, HA-STUB1, and Myc-TOR3A plasmids. Flag-tagged RIG-I was immunoprecipitated and subjected to MS analysis.

**Table S1 Demographic and clinical characteristics of Bronchiolitis patients and controls**

| Variables | Controls | Bronchiolitis | *P* |
| --- | --- | --- | --- |
| No. of subjects | 40 | 70 | N/A |
| Age, yrs | 1.21 ± 0.40 | 1.12 ± 0.47 | 0.35 |
| Male, n (%) | 16 (40.00%) | 39 (55.71%) | 0.11 |
| Clinical characteristics | | | |
| Cough, n (%) | N/A | 66 (94.28%) | N/A |
| Fever, n (%) | N/A | 43 (61.43%) | N/A |
| Wheezing, n (%) | N/A | 50 (71.43%) | N/A |
| Breathlessness, n (%) | N/A | 18 (25.71%) | N/A |
| dyspnea, n (%) | N/A | 16 (22.86%) | N/A |
| Grade | | | |
| Mild, n (%) | N/A | 48 | N/A |
| Moderate, n (%) | N/A | 20 | N/A |
| Severe, n (%) | N/A | 2 | N/A |

Table S2. Mice TOR3A gRNA sequences used for CRISPR/Cas9 technology

| Gene | Sequence (5′-3′) |
| --- | --- |
| Mice Tor3a gRNA1 | ATGCTATGAGATGGCAAGAT AGG |
| Mice Tor3a gRNA2 | GGGAGAATCAGAGGGGGGCC GGG |

Table S3. Mouse Stub1 gRNA sequences used for CRISPR/Cas9 technology

| Gene | Sequence (5′-3′) |
| --- | --- |
| Mouse Stub1 sgRNA1 | CGTGGGCCGCAAGTACCCGG |
| Mouse Stub1 sgRNA2 | GGGCCGCAAGTACCCGCGG |
| Mouse Stub1 sgRNA3 | CGGCGCCATGAAGGGCAAGG |

Table S4. Human TOR3A gRNA primer sequences used for CRISPR/Cas9 technology

| Gene | Sequence (5′-3′) |
| --- | --- |
| Human TOR3A sgRNA1-F | CACCGAAGAGCGTCCAGTACCGCT |
| Human TOR3A sgRNA1-R | AAACAGC GGTACTGGACGCTCTTC |
| Human TOR3A sgRNA2-F | CACCGCGTCCAGTACCGCTTGGAG |
| Human TOR3A sgRNA2-R | AAACCTCCAAGCGGTACTGGACGC |
| Human TOR3A sgRNA3-F | CACCGCGCCACGGACCGCGAAGCA |
| Human TOR3A sgRNA3-R | AAACTGCTTCGCGGTCCGTGGCGC |

Table S5. List of primers used for qPCR analysis.

| Gene | Sequence (5′-3′) |
| --- | --- |
| 18s RNA Forward primer | CGGCTACCACATCCAAGGAA |
| 18s RNA Reverse primer | GCTGGAATTACCGCGGCT |
| MurineIfnb1 Forward primer | ATGAGTGGTGGTTGCAGGC |
| Murine Ifnb1 Reverse primer | TGACCTTTCAAATGCAGTAGATTCA |
| Murine Tor3a Forward primer | TGCTTAAGGCTGGATGGTCCAGGGA |
| Murine Tor3a Reverse primer | CATCTCGGACGCACAGC |
| RSV-F Forward primer | GAATTGCAGTTGCTCATGCAA |
| RSV-R Reverse primer | TGGCGATTGCAGATCCAACA |
| RSV-M2-1 Forward primer | AAGTGGAGCTGCAGAGTTGG |
| RSV-M2-1 Reverse primer | GTGAGGAGTTTGCTCATGGC |
| RSV-N Forward primer | CATCTAGCAAATACACCATCCA |
| RSV-N Reverse primer | TTCTGCACATCATAATTAGGAGTATCAA |
| VSV-G Forward primer | ACGGCGTACTTCCAGATGG |
| VSV-G Reverse primer | CTCGGTTCAAGATCCAGGT |
| Human IFNB1 Forward primer | GCTCATGGAAAGAGCTGTAGTG |
| Human IFNB1 Reverse primer | ATGACCAACAAGTGTCTCCTCC |
| Human ISG15 Forward primer | GGGACCTGACGGTGAAGATG |
| Human ISG15 Reverse primer | CGCCGATCTTCTGGGTGAT |
| Human TOR3A Forward primer | TTAGAGTGGGACCTGAATGT |
| Human TOR3A Reverse primer | TGGGGTGAGGAAAGTGG |
| Human RIG-I Forward primer | CACCTCAGTTGCTGATGAAGGC |
| Human RIG-I Reverse primer | GTCAGAAGGAAGCACTTGCTACC |
