# Supplementary figures and images for: TOR3A represses type I interferon production and limits viral clearance during respiratory syncytial virus infection
Source: Emerg Microbes Infect. 2026 Feb 25;15(1):2637961. doi: 10.1080/22221751.2026.2637961 (PMC12990277; doi:10.1080/22221751.2026.2637961)

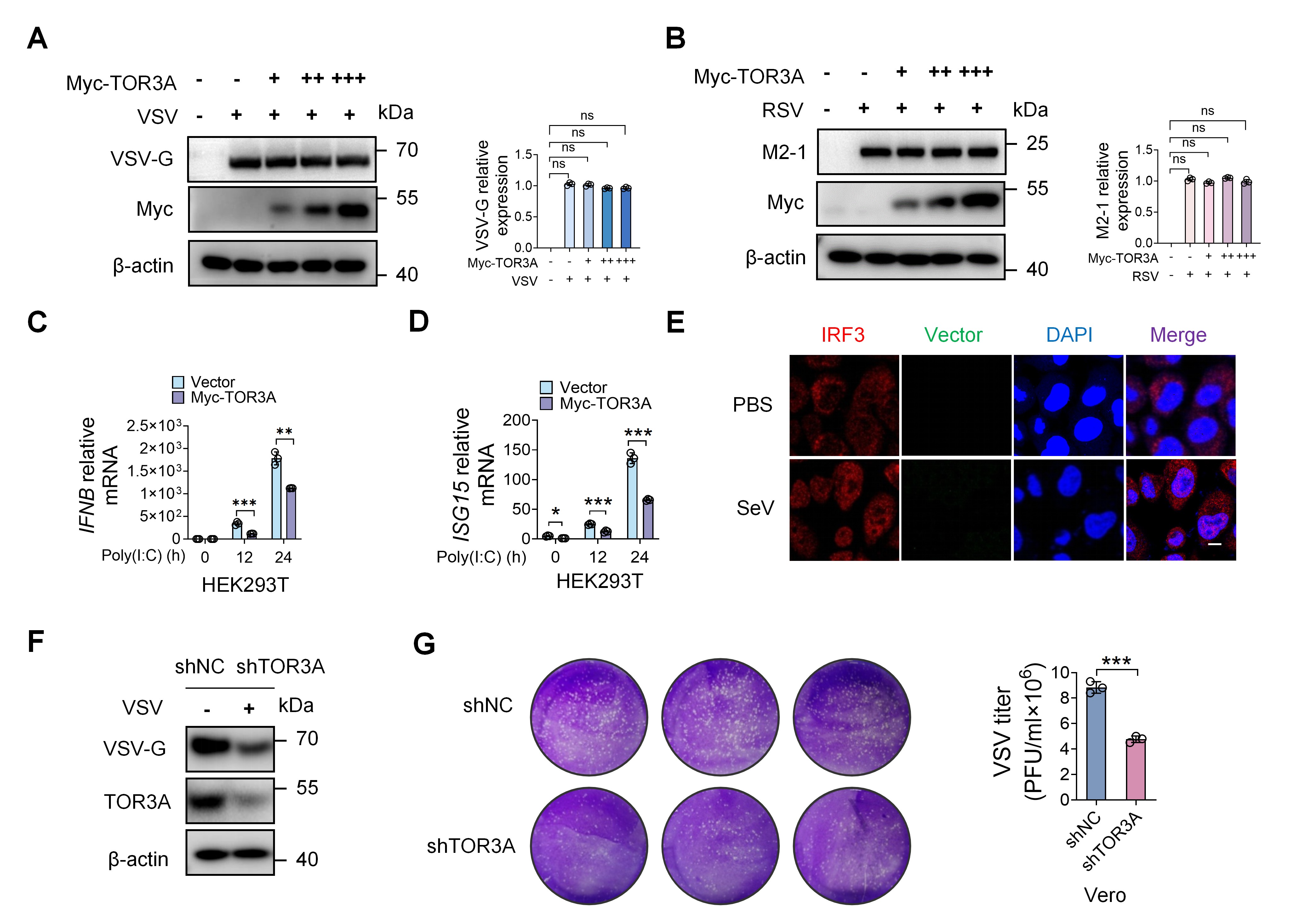

Supplement: FigS3.jpg [file TEMI_A_2637961_SM9108.jpg]

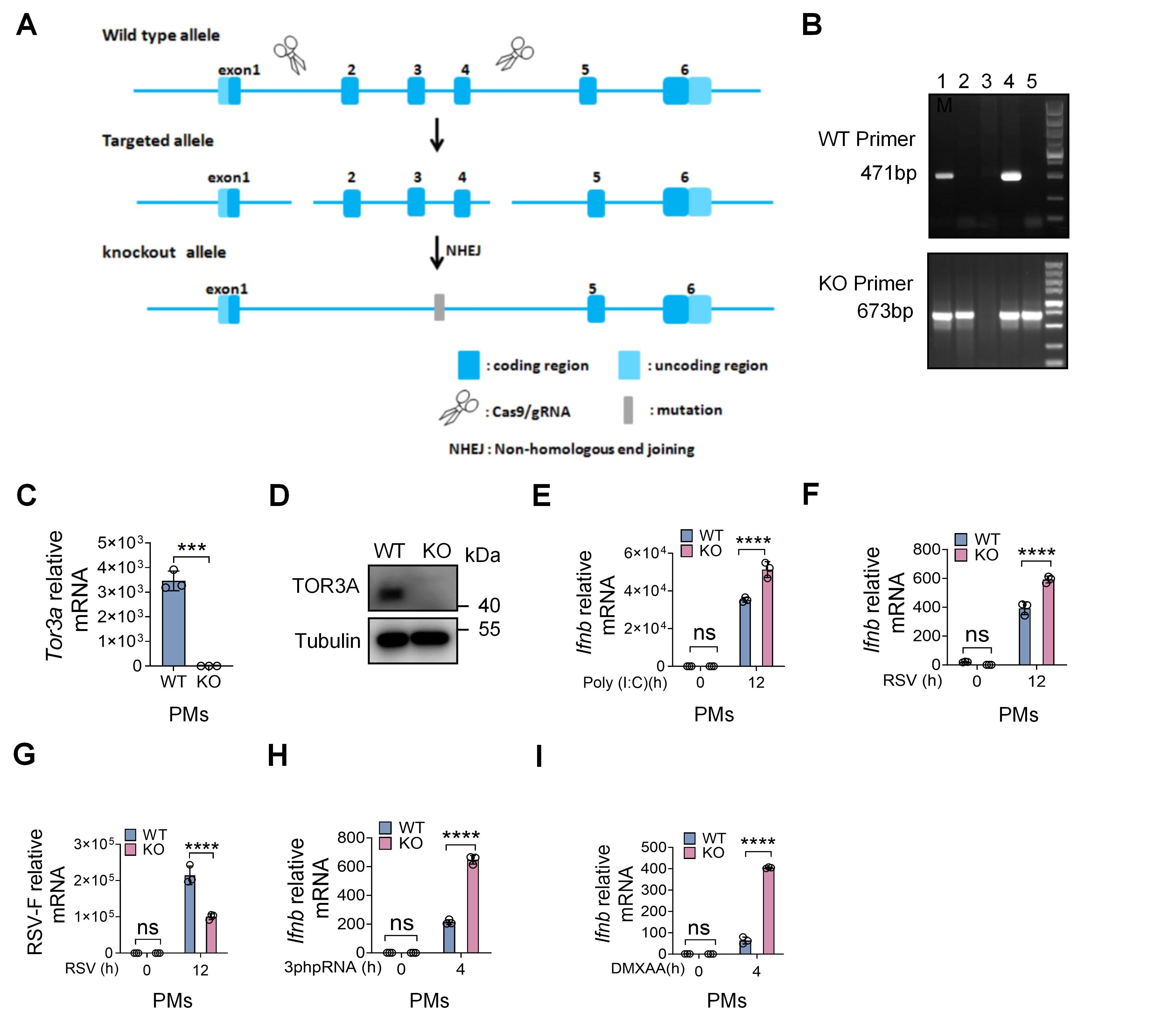

Supplement: FigS4.jpg [file TEMI_A_2637961_SM9107.jpg]

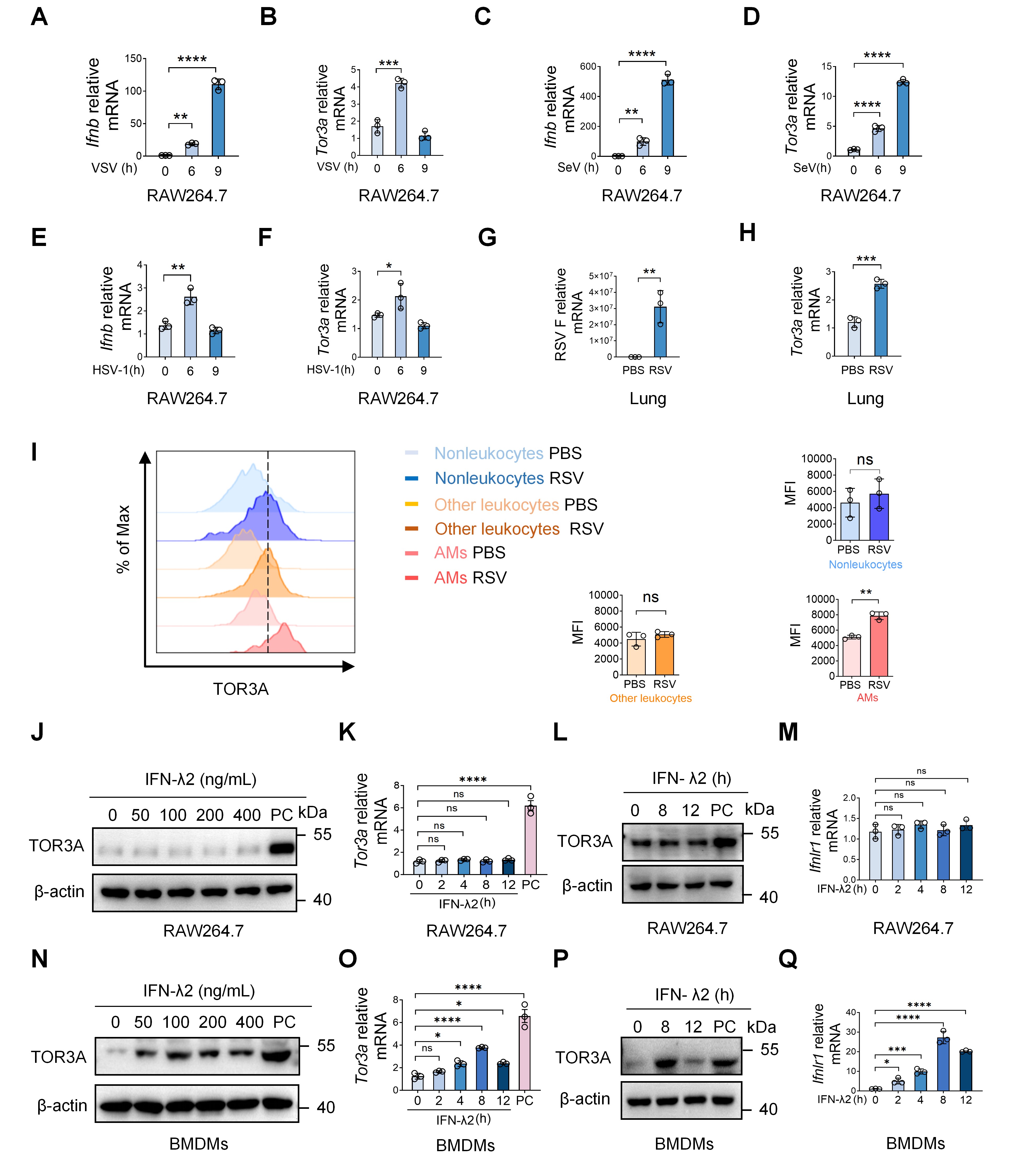

Supplement: FigS2.jpg [file TEMI_A_2637961_SM9106.jpg]

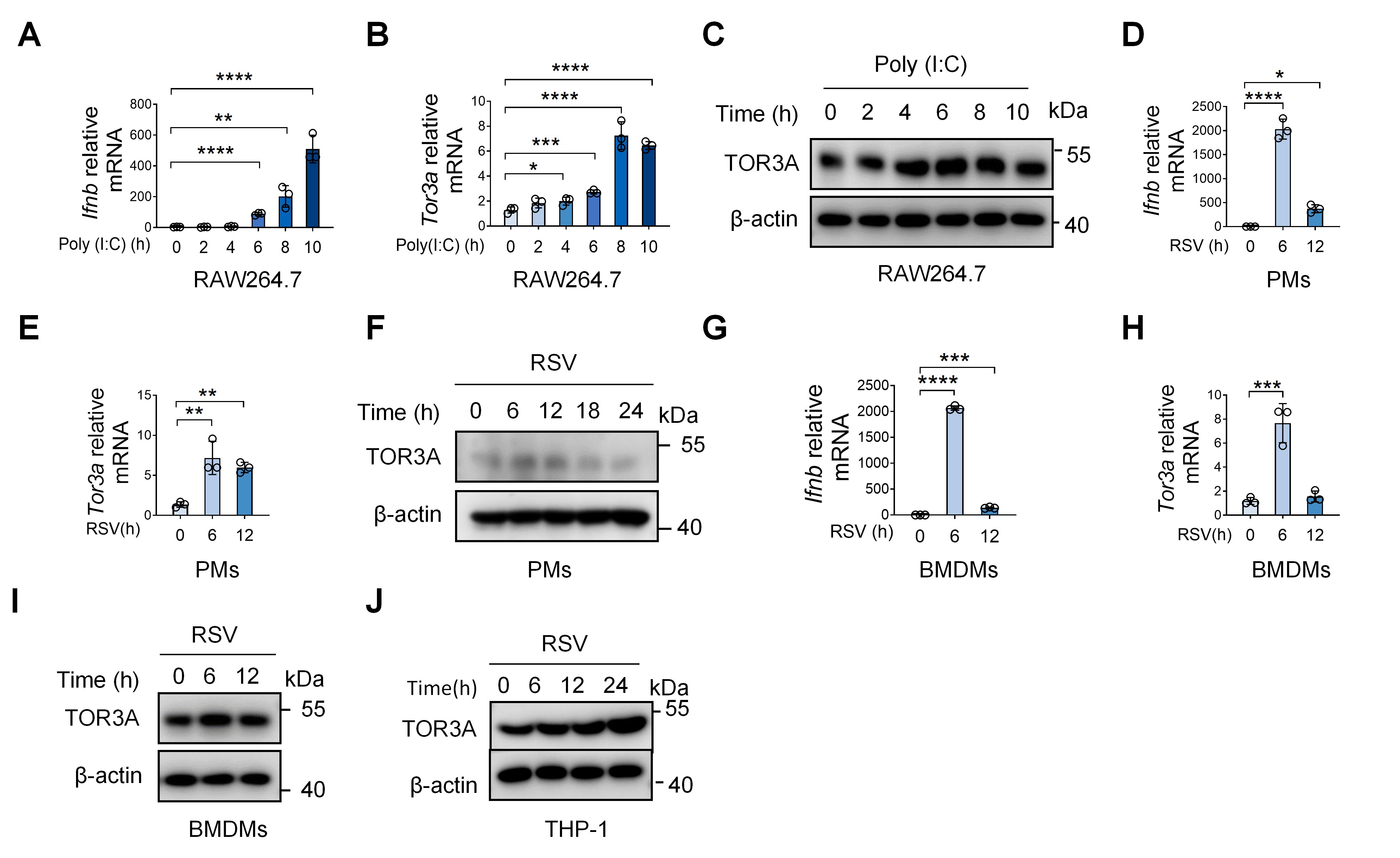

Supplement: FigS1.jpg [file TEMI_A_2637961_SM9105.jpg]

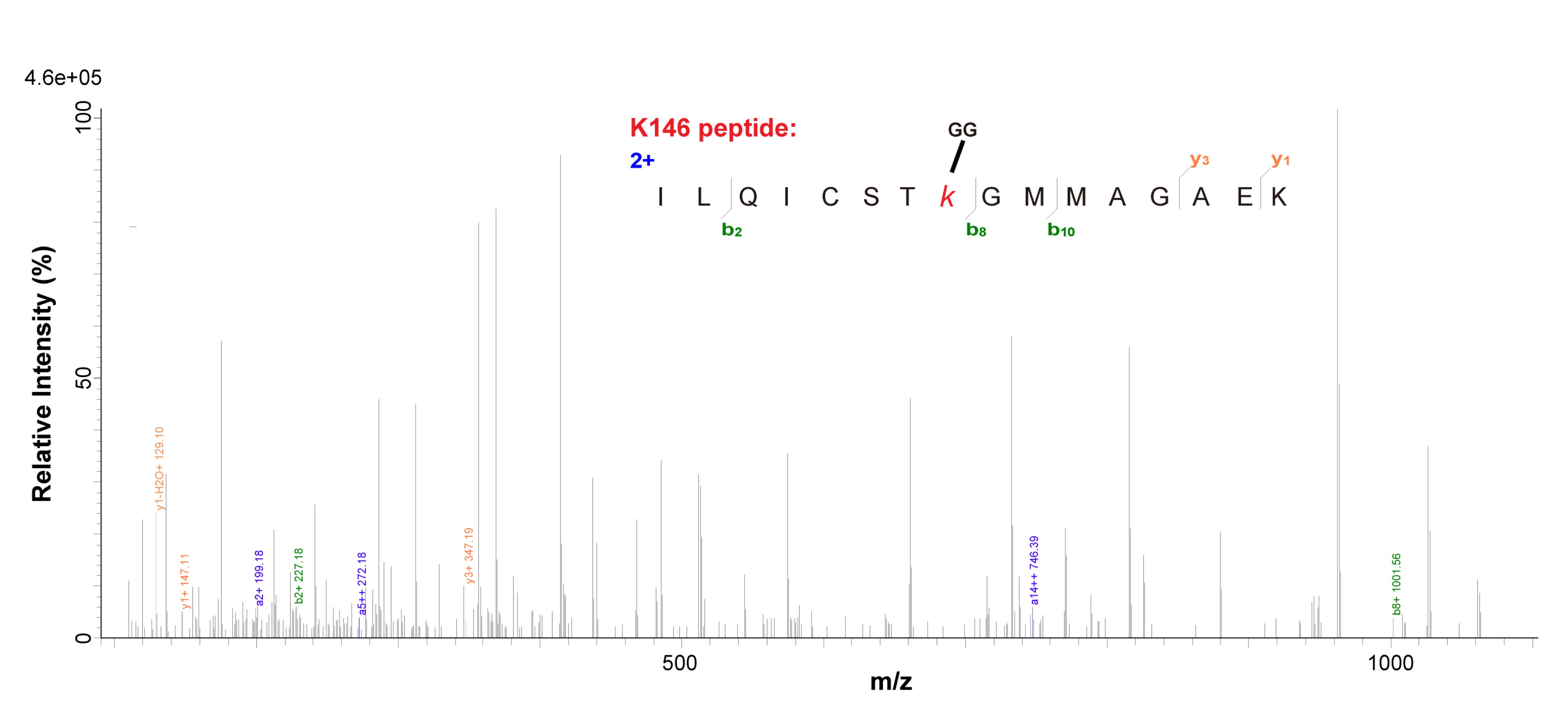

Supplement: FigS5.jpg [file TEMI_A_2637961_SM9104.jpg]
